# Chromatin states modify network motifs contributing to cell-specific functions

**DOI:** 10.1038/srep11938

**Published:** 2015-07-14

**Authors:** Hongying Zhao, Tingting Liu, Ling Liu, Guanxiong Zhang, Lin Pang, Fulong Yu, Huihui Fan, Yanyan Ping, Li Wang, Chaohan Xu, Yun Xiao, Xia Li

**Affiliations:** 1 College of Bioinformatics Science and Technology, Harbin Medical University, Harbin 150081, China; 2 Key Laboratory of Cardiovascular Medicine Research, Harbin Medical University, Ministry of Education.

## Abstract

Epigenetic modification can affect many important biological processes, such as cell proliferation and apoptosis. It can alter chromatin conformation and contribute to gene regulation. To investigate how chromatin states associated with network motifs, we assembled chromatin state-modified regulatory networks by combining 269 ChIP-seq data and chromatin states in four cell types. We found that many chromatin states were significantly associated with network motifs, especially for feedforward loops (FFLs). These distinct chromatin state compositions contribute to different expression levels and translational control of targets in FFLs. Strikingly, the chromatin state-modified FFLs were highly cell-specific and, to a large extent, determined cell-selective functions, such as the embryonic stem cell-specific bivalent modification-related FFL with an important role in poising developmentally important genes for expression. Besides, comparisons of chromatin state-modified FFLs between cancerous/stem and primary cell lines revealed specific type of chromatin state alterations that may act together with motif structural changes cooperatively contribute to cell-to-cell functional differences. Combination of these alterations could be helpful in prioritizing candidate genes. Together, this work highlights that a dynamic epigenetic dimension can help network motifs to control cell-specific functions.

Epigenetics has become one of the most promising and expanding fields in current biological researches. Diverse post-translational modifications in the tails of histone proteins have been validated to exert important functions in modulating gene expression and be involved in many biological processes, such as development and cell proliferation^1^. Distinct histone modifications can give rise to active or repressed states of key regulatory elements, such as H3K4me3-marked active promoters and H3K27me3-marked silent regions, contributing to regulation of gene expression. Such properties of epigenetic marks have been successfully used to comprehensively identify various regulatory elements through characterizing chromatin states across the human genome^2^. Accumulating evidence further indicates that regulatory elements marked by different epigenetic modifications can lead to open or closed chromatin conformations, thereby regulating the accessibility of regulatory elements and influencing transcription factor (TF) binding^3^. In parallel, recent studies also revealed that TF binding can accompany specific chromatin state changes by the recruitment of chromatin modification complexes.

A limited cohort of TFs regulating a large variety of targets form complex transcriptional regulatory networks for precisely and globally organizing gene expression^4^. Extensive studies have demonstrated that a small set of circuits exhibit much higher frequencies than expected at random. Such recurring circuits in regulatory networks have been termed network motifs. One of the most important network motifs is feedforward loop (FFL), in which a primary TF regulates a secondary one, and both target a final gene. FFLs play important roles in regulation of most cellular pathways.

Thus, we assume that specific chromatin modifications can influence FFL regulation, and subsequently contribute to biological functions. To address this hypothesis, we constructed chromatin state-modified regulatory networks in which nodes were labeled with different chromatin states. We searched for significant chromatin state-modified network motifs in different cell types and investigated their expression-, dynamic- and function-related properties. We found that FFLs coupled with diverse chromatin states were highly cell selective, and were associated with maintenance of cell-specific functions. We also found that cell-cell differences were partly dependent on specific chromatin state changes in specific types of motifs. Our results suggest that chromatin states appear indispensable for insights into how network motifs are involved in transcription regulation. Based on the important roles of chromatin states in network motifs, integration of chromatin states and structures of motifs allowed us to prioritize candidate genes for their contribution to cancers.

## Results

### Revealing transcriptional regulatory networks modified by chromatin states

In order to explore how chromatin states modify network motifs, we constructed transcriptional regulatory networks in four cell lines, consisting of H1, GM12878, K562 and HepG2, through the combination of 269 ChIP-seq data sets and DNase I hypersensitive sites (DHS) (see Methods).

**Figure 1:**
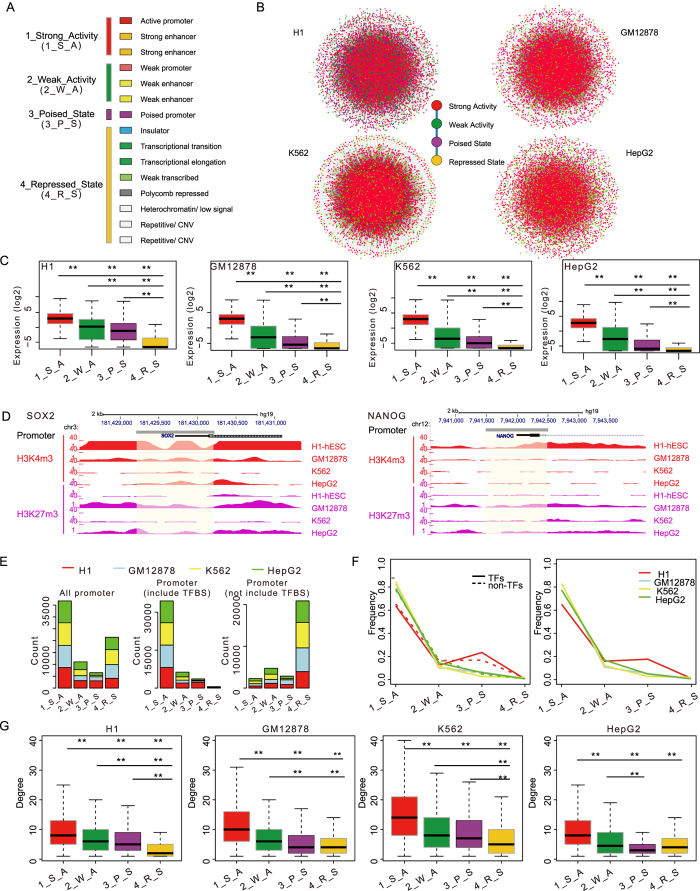
Detailed information of regulatory networks and their associated chromatin states. (**A**) Four broad chromatin states used in this study from fifteen chromatin states by Ernst *et al*. (**B**) The chromatin-state modified networks in four cell lines. The colors represent different chromatin states. (**C**) The log2 expression level of targets in FFL coupled with different chromatin states. (**represents *P*-value < 1.1e-13 from Wilcoxon rank-sum test; colors correspond to chromatin states). (**D**) H3K4me3 (red) and H3K27me3 (purple) occupancy at the *SOX2* and *NANOG* promoter regions in four cell lines. (**E**) The distribution of chromatin states for all promoters, one with TF binding and one without. (**F**) The frequency of genes with different chromatin states in four cell lines (solid for TFs and dotted for non-TFs in left panel; all genes in right panel). (**G**) The degree distribution of genes for each chromatin state in four cell lines (**represents *P*-value < 0.01 from Wilcoxon rank-sum test; colors correspond to chromatin states).

Considering chromatin states of nodes (TFs and targets) in different cell lines, we obtained genome-wide maps of 15 chromatin states, which were used for systematic annotation of the human genome in^2^, and sought to classify them into different categories. In order to determine the optimal number of chromatin state categories, we used seven histone modifications from ENCODE project (H3K4me1, H3K4me2, H3K4me3, H3K27ac, H3K27me3 and H3K9me3 over the promoter, and H3K36me3 over coding region) to characterize genes across four cell lines. The seven-dimensional histone modification profiles (Reads Per Kilobase per Million mapped reads (RPKM) values) from four cell lines were concatenated. The gap statistic (‘clusGap’ function in R package) was used to determine the optimal number of chromatin state categories by comparing observed within-cluster dispersion with its expectation. We observed that the maximum gap value is observed at 4 (Supplementary Fig. S1A). Besides, accumulating evidence have established some epigenetic states contributing to different expression levels^2^, including the active state marked by H3K4me1, H3K4me2, H3K4me3, H3K27ac but not H3K27me3, the weak activity state by moderate level of the active histone modifications, the repressed state by H3K27me3 or H3K9me3 but not H3K4me3, and the poised state by H3K4me3 and H3K27me3. We thus grouped these states into four broad categories including strong activity, weak activity, poised state and repressed state (Fig. 1A). In detail, ‘Active promoter’ and ‘Strong enhancer’ which marked by active epigenetic marks such as H3K4me1, H3K4me2, H3K4me3, H3K27ac but not H3K27me3 were combined as ‘Strong activity’ state. These active epigenetic marks have been reported to show high levels at promoters of high expressed genes^5^. Recently, weak chromatin state (such as weak promoter) was frequently studied^2^,^6^. They are characterized by moderate levels of active histone modifications and associated with intermediate expression levels. ‘Weak promoter’ and ‘Weak enhancer’ which marked by moderate levels of active epigenetic marks such as H3K4me1, H3K4me2 and H3K4me3 were thus combined as ‘Weak activity’ state. ‘Poised promoter’ state characterized by both the H3K4me3 and H3K27me3 marks is regarded as ‘Poised state’^7^, which plays important roles in cell differentiation^8^. These states without obvious enrichment of active epigenetic marks such as H3K4me1, H3K4me3 and H3K27ac were grouped into ‘Repressed state’ (such as ‘Polycomb repressed’, ‘Heterochromatin/low signal’ and ‘Insulator’). These agree with our results that various epigenetic marks (such as H3K27ac and H3K27me3) showed significant difference between different chromatin state categories (Supplementary Fig. S1B, S1C). Then, we identified chromatin states for each gene in regulatory networks by enrichment of epigenetic states at the promoter (see Methods). Finally, for each cell type, a chromatin state-modified transcriptional regulatory network was constructed (Fig. 1B). The number of regulatory interactions ranged from 94,509 to 151,589 and networks exhibited scale-free power-law degree distributions (Supplementary Fig. S2 and Supplementary Table S1).

In accordance with chromatin states having a previously described role in gene expression, we found that genes with different chromatin states showed differential expression (*P*-values < 1.1e-13, Wilcoxon rank-sum test, Fig. 1C and Supplementary Fig. S3) in four cell lines. For example, core TFs *SOX2* and *NANOG* show particularly high expression in hESC, and they showed enrichment of H3K4me3 and loss of H3K27me3 when compared with other cell types (Fig. 1D). Genes with/without TF binding sites in the promoters were highly enriched with active/silent chromatin states (Fig. 1E). Besides, we found similar distributions of chromatin states between TF and non-TF genes and between different cell lines except H1 (Fig. 1F). Interestingly, degrees of gene with different chromatin states consistently showed significant differences in all cell lines (Fig. 1G), suggesting a close relationship between chromatin states and topological structures.

### Identifying chromatin state-modified network motifs

We sought to systematically search for three-node motifs by taking into account both the topological structures of networks and chromatin states of nodes. Comparing to random networks, we then detected over-represented three-node motifs coupled with chromatin states, which were defined as chromatin state-modified network motifs.

**Figure 2:**
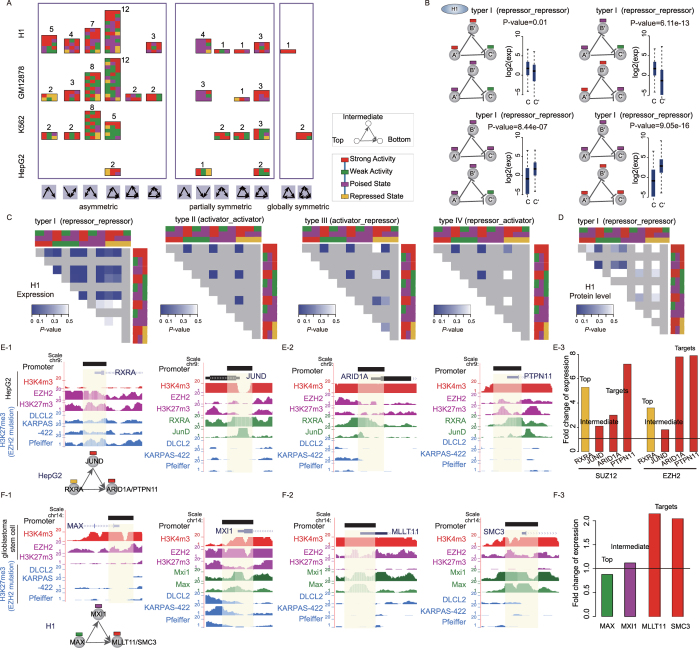
The landscape of chromatin state-modified motifs and their contribution to target expression. (**A**) Over-represented chromatin state compositions associated with network motifs in four cell lines. Asymmetric, partially and globally symmetric motifs are shown from left to right. For the symmetric motifs, only one of possible combinations of chromatin states was displayed. Chromatin states of gene promoter in each type of motif are shown in the order of top, intermediate and bottom positions. Values are presented as the number of chromatin state compositions. (**B**) Examples of FFLs in type I (both of top and intermediate TFs having repressive effects on their targets) can lead to expression differences of target genes (Wilcoxon rank-sum test). (**C,D**) Significant differences in expression, protein levels^69^ of targets between different chromatin state compositions within type I FFLs in H1 cell line. Color intensities (blue) correspond to *P*-values of Wilcoxon rank-sum test results. Gray entries represent data unavailable. Colored rectangles indicated the chromatin states of genes at top, intermediate and bottom position in FFLs. (**E**) Two examples of FFLs before and after knockdown of *EZH2* in HepG2. Promoters are analyzed for the distribution of histone modifications (such as H3K27me3 levels before and after EZH2 mutation in HepG2 and DLBCL cell lines: DLCL2, KARPAS-422 and Pfeiffer) and TF binding signal. E-3 shows fold-change expression of FFL instances after *EZH2* knockdown (colors of bar indicate their chromatin states). (**F**) Two examples of FFLs in H1 cell line after *EZH2* knockdown in GBM stem cell. Promoters are analyzed for the distribution of histone modifications and TF binding signal. F-3 shows fold-change expression of FFL instances after *EZH2* knockdown (colors of bar indicate their chromatin states).

We found a number of significant chromatin state-modified motifs in the four cell types, including 41 in H1, 36 in GM12878, 26 in K562 and 5 in HepG2, referring to a total of twelve types of motif structures (Fig. 2A). FFL, one of the most important network motifs, was linked with multiple chromatin state compositions and was consistently present in four cell types. The fully open states (i.e., all nodes in FFL are strongly activated) were found to be the most enriched state composition in H1 and K562, suggesting its fundamental role in FFL (Supplementary Fig. S4A). Also, we found that other significantly enriched chromatin state compositions tend to show changes of chromatin states in more than one position (top, intermediate or bottom) when comparing with the major fully open states (Supplementary Fig. S4B).

Chromatin modification acts as a factor contributing to expression fluctuations of TFs, which can propagate through regulatory interactions^9^ and in turn influence downstream targets. Thus, it is reasonable to assume that chromatin states of both targets and their upstream regulators can influence the expression of targets by network motifs. To address the hypothesis, we analyzed expression levels of target genes in instances of chromatin state-modified FFLs. We removed these targets shared by different types of state-modified FFLs to avoid combination of control^10^. Notably, the edge directions of FFLs represent active or repressive effects of TFs on their targets. We thus manually searched PubMed and obtained 29 and 28 TFs that can act as activators and repressors, respectively (Supplementary Table S2). Activators (or repressors) are regarded to play active (or repressive) effects on all of their targets. Then, we classified FFLs into four different types based on the active or repressive effects of TFs (type I: top and intermediate TFs are repressors; type II: top and intermediate TFs are activators; type III: top TF is an activator and intermediate TF is a repressor; type IV: top TF is a repressor and intermediate TF is an activator). Interestingly, in type I FFLs, we observed significant differences in expression of target genes between some specific chromatin state compositions, even between those with the same chromatin states of targets (Wilcoxon rank-sum test, Fig. 2B). For example, in H1 cell line, the target genes expression of two chromatin state-modified FFLs (one is ‘strong activity’ state at top- and intermediate positions, and ‘weak activity’ state at bottom position; the other is ‘poised’ state at top- and intermediate positions and ‘weak activity’ state at bottom position) showed significant difference (*P*-value = 0.01). Subsequently, we analyzed all types of comparable FFLs in four cell lines and found significant expression difference between different chromatin state compositions (Fig. 2C and Supplementary Fig. S5). In addition, for each type of comparable FFLs, we also found significant differences in protein abundance and phosphorylation level of target genes between different chromatin state compositions (Fig. 2D and Supplementary Fig. S5).

**Figure 3:**
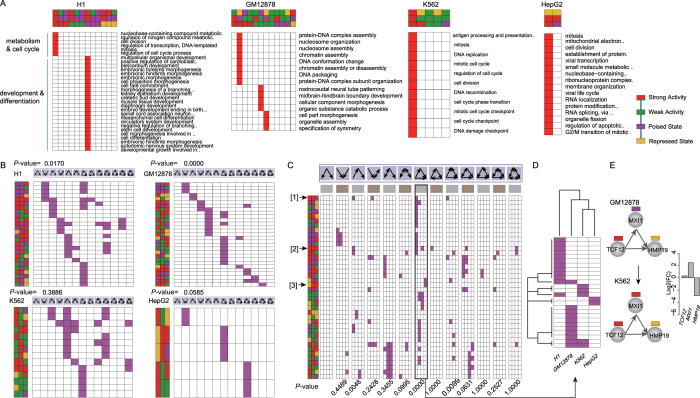
Specific chromatin state-modified network motifs and their specific biological functions. (**A**) The significant biological processes enriched by target genes of each chromatin state-modified FFL. The distribution of chromatin state compositions across thirteen types of motifs (**B**) in each cell line and (**C**) across four cell types. The *P*-values were obtained using mutual exclusivity test (see Methods). (**D**) The two-way clustering of state profile of chromatin state-modified FFLs across four cell lines. (**E**) An examples of changes of chromatin state-modified FFLs in the comparison between GM12878 and K562. The log2-transformed fold changes in gene expression are showed.

Besides, we sought to further investigate whether alteration of chromatin states of upstream regulators can induce the expression changes of target genes by knockdown of *EZH2*, an H3K27me3 methyltransferase, which can result in decreased H3K27 trimethylation. Perturbation of H3K27me3 would be expected to influence expression of repressed genes (with poised or repressed chromatin state) but not for active genes (with strong or weak activity state). Thus, we focused on FFL instances with poised or repressed chromatin states at the upstream regulators and activity states at the target genes to avoid the influence of the epigenetic state of target genes. By analyzing genome-wide expression of *SUZ12* and *EZH2* shRNA (GSE54108) in HepG2, we found that many FFL instances showed obvious expression differences of target genes (Fig. 2E). Two FFL examples, *RXRA*-*JUND*-*ARID1A* and *RXRA*-*JUND*-*PTPN11*, are coupled with repressed state of *RXRA* (top TF), strong activity of *JUND* (intermediate TF, Fig. 2E-1) and two targets *ARID1A* and *PTPN11* (Fig. 2E-2). Knockdown of *EZH2* or *SUZ12* led to increased expression of *RXRA* (fold-change = 5.3 for *SUZ12* and 3.6 for *EZH2*), and *JUND*, a tumor suppressor^11^ (fold-change = 2.1 for *SUZ12* and 1.8 for *EZH2*). Notably, the expression of target genes *ARID1A* and *PTPN11*, two tumor suppressors^12^, ranged from 3.0 to 7.9 fold increase (Fig. 2E-3). Furthermore, we analyzed H3K27me3 levels before and after *EZH2* mutation in HepG2 and three diffuse large B-cell lymphoma (DLBCL) cell lines (GSE40970), respectively. We found that the top TF *RXRA* showed a strong decrease of H3K27me3 levels after *EZH2* mutation, yet the intermediate TF *JUND* and targets *ARID1A* and *PTPN11* showed similar H3K27me3 levels before and after *EZH2* mutation (Fig. 2E). These findings highlight the importance of chromatin state of upstream regulators on target gene expression. Additionally, by analyzing another data of *EZH2* shRNA in human glioblastoma stem cell (GSE18150), we observed similar results. *MAX*-*MXI1*-*MLLT11* and *MAX*-*MXI1*-*SMC3* are coupled with weak activity of *MAX* (top TF, a partner protein of proto-oncogene *MYC*), poised state of *MXI1* (intermediate TF, a transcriptional repressor of *MYC*^13^ (Fig. 2F-1) and strong activity of two targets *MLLT11* and *SMC3* (Fig. 2F-2). After knockdown of *EZH2*, the expression of *MXI1* slightly increased (fold-change = 1.2). Importantly, the target genes *MLLT11* and *SMC3* showed more than 2-fold increase in expression (Fig. 2F-3). These observations may suggest that diverse chromatin states are exploited by FFLs to finely regulate gene expression.

### Cell specificity of chromatin state-modified FFLs for maintenance of cell-specific functions

To explore how these chromatin state-modified motifs affect biological functions, we performed functional enrichment analysis on target genes of each chromatin state-modified FFL in the four cell types (FDR < 0.05). We found that the targets were significantly involved in many important biological functions, such as DNA damage checkpoint and cell cycle. Strikingly, these functions showed a mutually exclusive pattern across different chromatin state compositions in all cell types (Fig. 3A). For instance, in H1, the chromatin state composition (top-, intermediate- and bottom positions with ‘strong activity’ states) could capture functions associated with mitosis and metabolism, however, another chromatin state composition (top and intermediate positions with ‘strong activity’, and bottom position with ‘poised state’) was related to development and differentiation. High functional specificity of chromatin state-modified motifs emphasizes an important role of various distinct chromatin states in motif-mediated maintenance of cell homeostasis.

We next examined whether chromatin state compositions were commonly used by different motifs in a specific cell line. We found a large number of specific chromatin state compositions and only a few common ones shared by multiple motifs in H1 and GM12878 (*P*-value < 0.05, permutation test; see Methods; Fig. 3B), suggesting that different types of motifs are related to specific chromatin states, probably due to their distinct structural organization.

We further explored whether consistent chromatin states are coupled with the same types of motifs across different cell types. To our surprise, mutually exclusive patterns of chromatin states across different cell types were clearly evident for the FFL and ‘regulated mutual’ (both *P*-value < 0.01, permutation test; see Methods; Fig. 3C), suggesting that chromatin state-modified motifs are highly cell selective. Interestingly, the state profile of FFL had the ability to distinguish H1 and GM12878 from K562 and HepG2, when clustering four cell types based on the state profile (Fig. 3D). One chromatin state composition associated with FFL (top position with ‘strong activity’ state, intermediate position with ‘poised state’ and bottom position with ‘repressed state’) was observed both in H1 and GM12878, but not in K562 and HepG2. For example, in *TCF12*-*MXI1*-*HMP19*, the intermediate TF *MXI1*, a negative regulator of cell cycle^14^, showed a state transition from ‘poised state’ to ‘strong activity’ state in the comparison of GM12878 and K562. The epigenetic alteration of *MXI1* lead to its increased expression, and thus greatly reduce the expression of downstream target *HMP19* by *MXI1*-mediated inhibition (Fig. 3E). The downstream target *HMP19* has been verified to be a tumor/metastasis suppressor^15^. These findings further support that epigenetic changes of major upstream regulators play important roles in the expression of downstream targets.

**Figure 4:**
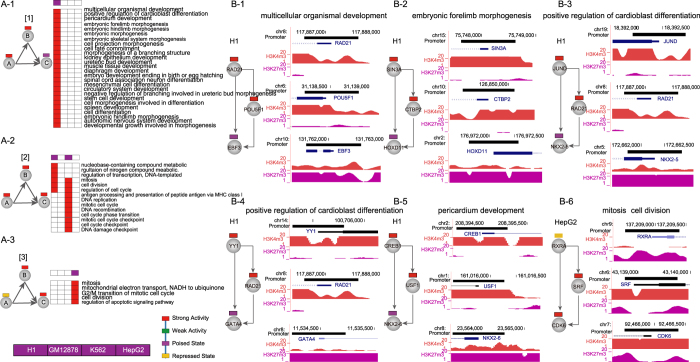
Examples of specific biological functions of cell-type-specific chromatin state-modified network motifs. (**A**) The significantly enriched biological processes with target genes in different types of chromatin state-modified FFL. The left of each panel shows the chromatin state-modified motifs. On the right, the purple rectangles represent whether a specific chromatin state composition is present (purple) or absent (white) in four cell lines. (**B**) Examples of chromatin-state modified FFL instances contributing to different biological processes and their associated H3K4me3 (red) and H3K27me3 (purple) distributions in H1 and HepG2.

**Figure 5:**
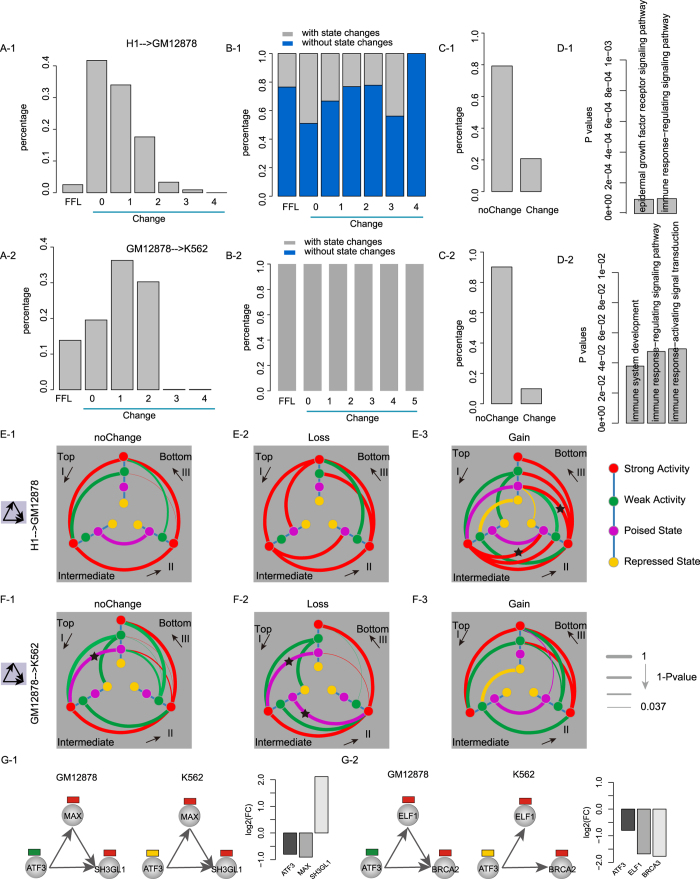
Changes of motif architecture and chromatin state in cell comparisons. (**A**) The percentage of changes in FFL architecture based on FFL instances in corresponding source cell line (such as H1 for H1-GM12878). The values of x-axis represent the number of edges of FFLs in the end cell types. (**B**) FFL instances in the source cell line were divided into different groups according to the alterations of FFL structures (including edge gain, loss or no change). For each group, the percentage of FFL instances with at least one chromatin state change under different types of changes of motif structures was shown. (**C**) The percentage of genes involved in FFL instances with chromatin state change or not. (**D**) The significant biological processes enriched by genes involved in FFL instances with chromatin state change. (**E**) Chromatin state transition of FFLs under different types of changes of motif structures: without alteration in motif structures (**E**-1; **F**-1), loss edges from FFL (**E**-2; **F**-2), gain edges (forming FFL) (**E**-3; **F**-3). The four types of chromatin states are arranged in the same order along each axis. The I, II and III quadrants separately represent chromatin state transitions at top-, intermediate-TF and target gene of a motif in an anticlockwise direction. Each curve represents a kind of chromatin state transition. Colors of curves indicate the chromatin states of genes at the starting point of comparison (e.g., H1 in H1-GM12878). The terminal points of curves represent chromatin states at the end point of comparison (e.g., GM12878 in H1-GM12878). The thickness of curves represents the significance of chromatin state transition by the value of 1-(*P*-value). (**G**) Examples of changes of chromatin state-modified FFLs. The log2-transformed fold changes in gene expression are showed.

Moreover, we analyzed functions enriched in target genes of chromatin state-modified FFLs in four cell lines and found that cell-specific chromatin state-modified motifs seem to be responsible for specific biological functions (Fig. 4). For instance, one H1-specific FFL coupled with ‘strong activity’ states (top and intermediate) and a ‘poised state’ (bottom) is associated with development (such as ‘multicellular organismal development’, ‘cell fate commitment’ and ‘pericardium development’) and cell differentiation (such as ‘positive regulation of cardioblast differentiation’) (Fig. 4A-1). As an example, *RAD21* cooperated with the master pluripotency gene *POU5F1* to regulate downstream the early B-cell factor 3 (*EBF3*). Both *RAD21* and *POU5F*1 playing important roles in maintaining hESC identity^16^ showed ‘strong activity’ states in H1. However, *EBF3* that had been implicated in B-cell differentiation, bone development and neurogenesis^17^ showed a ‘poised state’ (Fig. 4B-1), consistent with its essential role in stem cell maintenance and subsequent differentiation^18^. Another example was *SIN3A*-*CTBP2*-*HOXD11*, in which *SIN3A* (a repressor of pluripotency gene) with a ‘strong activity’ state directly regulated *CTBP2* (‘strong activity’) (Fig. 4B-2) which played an important role in maintaining a balance between self-renewal and differentiation^19^. And these TFs regulated *HOXD11*, which showed a ‘poised state’ in H1 and was required for limb development^20^. Similarly, *JUND*-*RAD21*-*NKX2-5* and *YY1*-*RAD21*-*GATA4* were associated with ‘positive regulation of cardioblast differentiation’ (Fig. 4B-3) and *CREB1*-*USF1*-*NKX2-6* was related to ‘pericardium development’ (Fig. 4B-5). These target genes, *NKX2-5*, *GATA4* and *NKX2-6*, have been reported to be essential for cardiac development^21^. These results are supported by previous studies that bivalent domains silenced lineage specific differentiation genes and loss of which was crucial for development^22^. We further used all poised genes of H1 network as background (referring to 3,064 genes) to perform functional enrichment analysis, to test whether the FFL target poised genes are selectively enriched in some specific developmental processes. We found that FFL target poised genes were selectively enriched in ‘negative regulation of apoptotic process’, ‘axon development’, ‘axonogenesis’ and ‘positive regulation of cell differentiation’ (FDR < 0.1; Supplementary Fig. S6A).

In addition, one type of H1 and K562-shared chromatin state-modified FFL (‘strong activity’ states at the top, intermediate and bottom, referred to as ‘all-strong-activity’ FFL) was associated with K562-related functions, such as ‘antigen processing and presentation of peptide antigen via MHC class I’ (Fig. 4A-2). For example, *SPI1*-*RFX5*-*(HLA-E)* was associated with ‘antigen processing and presentation of peptide antigen via MHC class I’, in which *SPI1* was a major factor for maintenance of germinal center B-cells^23^ and *HLA-E*, human histocompatibility leukocyte antigen, has been reported to be involved in MHC-I antigen processing and presentation pathways^24^ (Supplementary Fig. S7A). Also, we used used all ‘strong activity’ genes of K562 network as background (referring to 9,509 genes) to characterize specific biological processes of ‘strong activity’ genes of the ‘all-strong-activity’ FFL. The result showed that these ‘strong activity’ genes were selectively enriched in ‘antigen processing and presentation of exogenous peptide antigen via MHC class I, TAP-dependent’, ‘G2/M transition of mitotic cell cycle’ and ‘positive regulation of ubiquitin-protein ligase activity involved in mitotic cell cycle’ (FDR < 0.1; Supplementary Fig. S6B).

**Figure 6:**
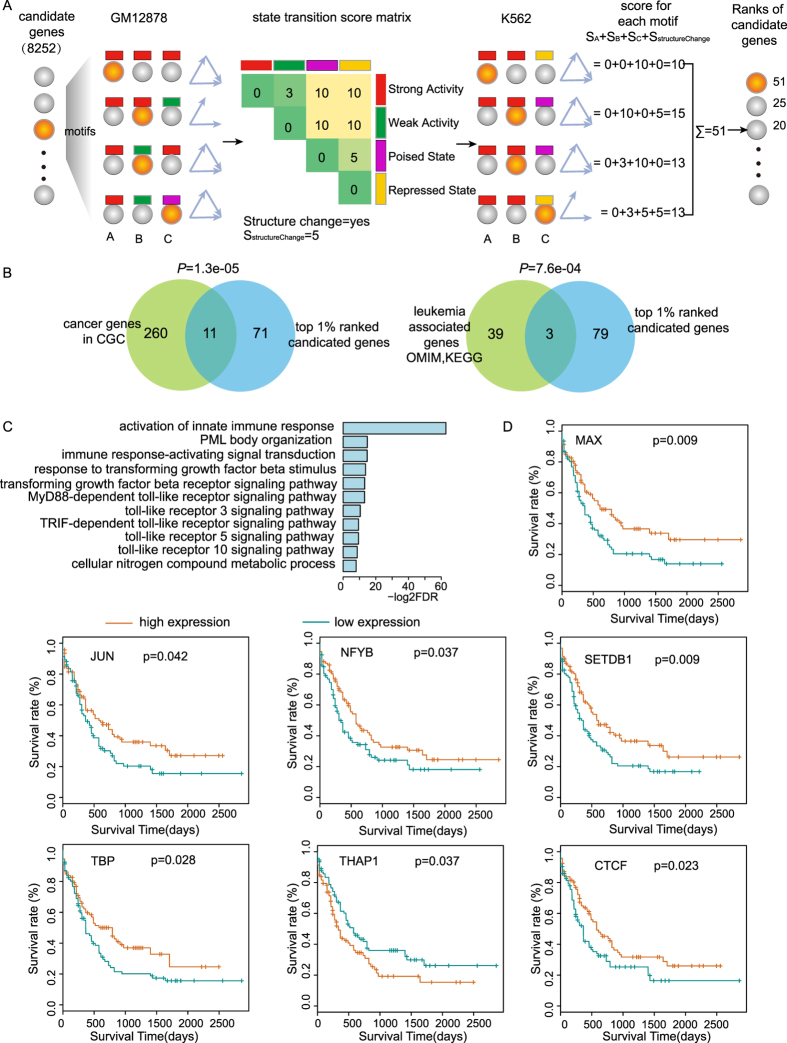
Prioritization of candidate genes by integrating changes of chromatin state and motif architecture. (**A**) The workflow based on a chromatin state transition matrix and structure alteration of FFLs for prioritization of candidate genes (see Methods). (**B**) The enrichment analysis between the top 1% of ranked genes and Cancer Gene Census (CGC) (*P*-value = 1.3e-05), leukemia-related genes derived from OMIM and KEGG databases (*P*-value = 7.6e-04). Statistical significance was calculated by hypergeometric test. (**C**) The GO biological processes enriched by the top 1% of ranked genes (FDR < 0.05). (**D**) Kaplan-Meier curves of the overall survival for high and low expression of top-ranked gene in 197 AML samples from TCGA. *P*-value was determined by log-rank test.

One type of HepG2-specific chromatin state-modified FFL, coupled with a ‘repressed state’ (top) and ‘strong activity’ states (intermediate and bottom), was associated with cancer-related functions, including mitosis and cell division (Fig. 4A-3). For instance, *RXRA* regulated *SRF*. They together regulated critical modulators of cell-cycle progression, including two members of *CDK* family (*CDK1* and *CDK6*), mitogen-activated protein kinase 1 (*MAP2K1*) and cell division cycle 25A (*CDC25A*) (Fig. 4B-6 and Supplementary Fig. S7). Taken together, these results consistently suggest that highly cell-selective state-modified network motifs are associated with maintenance of cell-specific functions and cell identity.

### FFLs with specific chromatin state changes contribute to cell-to-cell functional differences

We analyzed alterations of chromatin states and FFL structures in cell comparison (that is, from embryonic stem cell H1 to lymphoblastoid cell GM12878; from GM12878 to myelogenous leukemia cell K562) by extracting chromatin state-modified FFL instances. Unexpectedly, comparative analysis in H1-GM12878 and GM12878-K562 consistently showed that more than 85% FFLs (97.48% in H1-GM12878; 86.16% in GM12878-K562) architectures were changed (Fig. 5A). Chromatin state transitions were involved in 37.55% and 100.00% FFLs in H1-GM12878 and GM12878-K562, respectively (Fig. 5B). However, there were only 20.77% and 9.86% genes in FFL instances changed their chromatin states in H1-GM12878 and GM12878-K562, respectively (Fig. 5C). Furthermore, they were significantly enriched in immunity-related functions (e.g., immune response-regulating signaling pathway) in H1-GM12878 and GM12878-K562 (*P*-value < 0.05, Fig. 5D). Thus, we further analyzed chromatin state transitions in chromatin state-modified FFL with edge gain (or loss) or not. We observed that transition within ‘strong activity’ state was a high-frequency event (*P*-value < 1e-6 based on 1,000 permutation tests; see Methods; Fig. 5E, 5F). However, other chromatin state transitions in cell comparison were dependent on motif structure, edge gain or loss and cell types.

Notably, a remarkable chromatin state transition from the poised state to the strong activity state at the top position of FFLs without edge gain or loss (*P*-value < 1e-6) was observed in GM12878-K562 rather than in H1-GM12878 (Fig. 5E-1, black star). Another remarkable chromatin state transition from the poised state to the strong activity state at the top and intermediate positions of FFLs with edge loss (*P*-value < 1e-6) was observed in GM12878-K562 (Fig. 5E-2, black star). The finding was supported by a recent report that loss of H3K27me3 mark is a predictor of poor outcome in cancers^25^. Similarly, a chromatin state transition from the active state to the ‘poised state’ at the intermediate and bottom position (*P*-value < 1e-6) was unique to FFLs with edge gain in H1-GM12878 (Fig. 5E-3), suggesting a close association of state transitions with cell-to-cell differences.

In the *ATF3-MAX-SH3GL1*-formed FFL, *ATF3*, a tumor suppressor^26^, showed a state transition from ‘weak activity’ to ‘repressed state’ in the GM12878-K562 comparison. The epigenetic alteration results in its reduced expression level, and in turn increases the expression of *SH3GL1* by reducing *ATF3*-mediated inhibition of *SH3GL1*, which is a fusion partner in acute myeloid leukemia and plays a role in leukemogenesis. However, we did not observed any changes in the FFL architecture in the GM12878-K562 comparison (Fig. 5G-1). In the *ATF3-ELF1-BRCA2*, *BRCA2* (an anti-oncogene) intensely reduced their expression in K562 relative to GM12878, which together with loss of the regulation from *ELF1* to *BRCA2* (Fig. 5G-2). Consistently, aberration of *BRCA2* has been confirmed in acute myeloid leukemia^27^,^28^. Overall, our findings underscore their cooperative relationships in a cell-specific manner.

### Chromatin state and structure alterations of FFL contribute to discovery of cancer genes

Such chromatin state and structure changes of motifs seem to play crucial roles in maintaining cell-specific functions, which inspired us to integrate chromatin state and structure alterations of motifs for prioritization of candidate genes^29^ (Fig. 6A). By quantifying changes in chromatin state and FFL architecture between cancer (i.e., K562) and normal (i.e., GM12878) cells using the criteria described in Methods, in which poised and repressed state with suppression effects on gene expression were regarded as similar chromatin states; strong and weak activity state associated with consistent epigenetic marks with different intensity were regarded as proximate chromatin states. Chromatin state changes between activation (e.g., strong and weak activity states) and repression (e.g., poised and repressed states) seriously affected gene expression and thus were given by the highest score. And chromatin state changes within similar or proximate chromatin states were given by relatively low score. Finally, we assigned a summary score to each candidate gene based on all of its implicated FFL instances. Particularly, when we changed the scoring matrix, the results was little changed. Ranking candidate genes according to their summary scores (Supplementary Table S3), we found that the top 1% of genes (referring to 82 genes) were significantly enriched in known cancer genes (*P*-value = 1.3e-05, hypergeometric test, Fig. 6B) from Cancer Gene Census (CGC)^30^. Furthermore, the top 1% of genes showed strong enrichment for leukemia-associated genes (*P*-value = 7.6e-04, hypergeometric test, Fig. 6B), which were collected from OMIM and KEGG databases. Functional enrichment analysis of the top 1% of genes revealed many functions involved in leukemia development (Fig. 6C), such as ‘PML body organization’, ‘immune response-activating signal transduction’, and ‘transforming growth factor beta receptor signaling pathway’.

These top-ranked genes were composed of 67 ‘source-node’ genes (the TFs with ChIP-seq data available in the corresponding cell type) and 15 ‘non-source-node’ genes. Notably, we found that the known cancer genes in the top 1% of genes were from ‘source-node’ genes. Thus, we manually annotated the 15 ‘non-source-node’ genes and found that some of them have been demonstrated to be involved in human tumorigenesis. For example, *LAPTM4B* (lysosome-associated protein transmembrane 4 beta), a novel cancer-related gene, has been showed to be amplified and overexpressed in many human malignancies^31^,^32^. An uncharacterized gene *FAM133B* at chromosome 7q21.2 was recently identified as a novel gene fusion partner of *CDK6*, a regulator of G1/S cell-cycle progression, in T-cell acute lymphoblastic leukemia (T-ALL)^33^. *STEAP1B* was found to be overexpressed in prostate cancer and associated with a down-regulated lncRNA (*AC002480.5*) in Chronic Lymphocytic Leukemia (CLL)^34^. Focal amplification of *BRF2* in chromosome 8p12, a RNA polymerase III (Pol III) transcription initiation factor, is an early event in lung tumorigenesis through Pol III-mediated transcription^35^. The tumor protein D52-like 1 (*TPD52L1*) involved in cell proliferation and cell cycle control and its overexpression was found to be associated with human breast and prostate cancers^36^. Differential methylation of *C7orf63* was observed between two diffuse large B-cell lymphoma (DLBCL) subtypes^37^. *ENDOV*, the DNA repair enzyme endonuclease V, participates in DNA repair and helps to prevent mutations^38^,^39^. *GPR176* is a member of G-protein-coupled receptors (GPCRs), the deregulation of GPCRs has been associated with tumorigenesis^40^. Additionally, we obtained RNA-seq data set of 4,466 Cancer Genome Atlas (TCGA) tumors from 12 cancer types and 549 normal samples. For each cancer type, we identified differentially expressed genes using DEseq2^41^ (FDR < 0.05 and fold change > 2). Comparing to randomly selected genes, the ‘non-source-node’ genes were differentially expressed in more cancer types (*P*-values < 0.001; 1,000 permutation tests, Supplementary Fig. S8). Together, these top-ranked ‘non-source-node’ genes may be novel key cancer/leukemia genes.

In our results, the first-ranked gene *MXI1*, a transcriptional repressor of *MYC*, had been reported to be correlated with a poor clinical outcome in acute leukemia^42^ and the second-ranked gene *FOS* was reported as an important regulator and its increased expression was associated with adverse prognosis^43^. Furthermore, we used Kaplan-Meier curves and log-rank test to evaluate the effect of top-ranked genes on overall survival using 197 AML samples from The Cancer Genome Atlas (TCGA). There were seven genes (including *MAX*, *JUN*, *NFYB*, *TBP*, *THAP1*, *SETDB1* and *CTCF*) among the 30 top-ranked genes showed statistically significant associations with survival (*P*-value < 0.05, log-rank test, Fig. 6D). We next assessed whether the top-ranked genes show cell-specific expression. A total of 180, 134, 88 and 88 cell type-specific genes were identified in H1, GM12878, K562 and HepG2, respectively (see Methods). Among the top 1% genes, only three cell type-specific genes (*NFE2*, *STEAP1B* and *TPD52L1*) in K562, one (*ETS1*) in GM12878 and one (*GPR176*) in H1 were observed. These findings support that both chromatin states and structures of motifs are important for maintenance of the steady state of cells and highlight that combination of dysfunctional information about the alteration of chromatin states and structures of motifs in cancer may allow identifying cancer genes.

## Discussion

Although roles of chromatin modification and TF binding in regulation of gene expression have been studied, from the perspective of network architecture-based integration, the insights into regulation are largely unknown. We performed a systematic analysis of the relationship between network motifs and chromatin states. We integrated a multitude of ChIP-seq and RNA-seq data from ENCODE project and chromatin states defined by multiple epigenetic marks to construct chromatin state-modified regulatory networks in four cell lines. Analysis of network motifs revealed that diverse motifs coupling chromatin state compositions were over-represented in all cell lines.

Our results showed that diverse chromatin state-modified FFLs were associated with maintenance of diverse functions. Especially, a poised state at the bottom position of FFL was observed exclusively in H1. Such chromatin state-modified FFL was primarily involved in development-related functions. Prior studies indicated that the poised state had the ability to rapidly respond to later transcriptional activation signal for differentiation^44^. Therefore, we suspected that the poised state might cooperate with FFL exclusively in H1 in order to generate a rapid response during differentiation by making information-processing more efficient.

We also found substantial changes in the FFL architecture and relatively few changes in chromatin states in cell comparisons. Notably, a set of immune-related genes exhibited chromatin state changes, which may be used to help to reprogram regulatory networks in differentiated cell lines. Moreover, chromatin state transitions, to some extent, showed specific patterns during comparisons of different cells, further supporting high cell specificity of chromatin state-modified motifs. It is supported by the recent observations of tissue specificity of regulatory network and epigenetic modification^45^,^46^. The specific chromatin state changes may be required to adapt specific cellular functions, consistent with previous reports of the complex relationship between dynamic epigenetic landscape and genomic function^47^. Chromatin modifications affect TF binding by altering the local chromatin structure or providing specific binding surfaces^48^. Distinct chromatin environments are related with specific combinatorial regulation of TFs^3^,^49^. Besides, context-specific regulation of chromatin regulators may be another explanation for specific epigenetic landscape and distinct functions. For example, tissue-specific subunits of the SWI/SNF complex, a switch in *BAF45* and *BAF53* subunit plays an important role in transiting neural stem cells into postmitotic neurons^50^. Therefore, a dynamic interplay of chromatin state and transcriptional regulation might contribute to cell-specific utilization of chromatin state-modified FFLs in our results. Distinct cellular microenvironments may be one of reasons for formation of cell-specific chromatin state-modified motifs, like dressing network motifs with diverse chromatin states in different cellular contexts. A previous study reported that similar lifestyle can shape similar regulatory interactions^51^. A possible explanation is that similar environments can generate similar epigenetic modification which may play an important role in shaping network topology^52^, indicating that diverse chromatin states may provide an additional safeguard for maintaining or promoting ‘sign-sensitive delay’ or ‘pulse generator’ function of FFLs.

We used the fold enrichment method to characterize chromatin states of genes based on 200-base-pair intervals along the genome. Indeed, previous studies have proposed different methods to characterize epigenetic patterns. For example, by integrating multiple histone modifications, Larson and Yuan developed a hidden Markov model (HMM) approach to detect epigenetic patterns of genes, which can provide easily interpretable outcomes^53^. Therefore, we applied a four-state HMM approach to directly re-assign a chromatin state to each gene. In detail, a seven-dimensional histone modification profile corresponding to the RPKM values of epigenetic marks over the promoter (for H3K4me1, H3K4me2, H3K4me3, H3K9me3, H3K27ac and H3K27me3) and coding region (for H3K36me3) was used to characterize chromatin states of each gene in four cell lines (ChIP-seq data from ENCODE project). We found that genes with different chromatin states showed differential expression in each cell line (*P*-values < 2.2e-12, Wilcoxon rank-sum test; Supplementary Fig. S9). Moreover, we found a high consistency, with an average of 74.99%, in the comparison between results from the HMM approach and those from the fold enrichment method (82.55% for H1, 79.63% for GM12878, 64.97% for K562 and 72.82% for HepG2). Notably, recent studies highlighted that distal binding sites of TFs can also play important transcriptional regulatory roles^54^,^55^,^56^. Such distal binding sites often located in regions of open chromatin, detected as DNaseI hypersensitive sites (DHS)^57^ and showed enrichment for enhancer mark H3K4me1^58^,^59^. Therefore, we sought to identify chromatin state-modified motifs in H1 cell line by considering distal binding sites based on chromatin states from the HMM approach (see Supplementary methods and Supplementary Fig. S10). We identified 14,918 distal enhancer-mediated regulatory interactions in H1 based on the method described in^60^. By comparing to random networks, a total of 21 chromatin state-modified motifs were identified (*P*-value < 0.05 and Nreal– Nrand > 0.05Nrand; Supplementary Fig. S11). Among these significant motifs, 85.71% are consistent with the above results. Moreover, differential expressions of targets were observed between different chromatin state compositions associated with FFLs. Also, functional enrichment analysis showed a high consistency with the above results, such as development and differentiation functions enriched by FFLs (top and intermediate position with ‘strong activity’ states and bottom positions with ‘poised’ states). Similarly, we also observed a mutually exclusive pattern of chromatin states across different motif structures (Supplementary Fig. S11). These findings further support cell-specific functions of chromatin state-modified FFLs.

Together, we systematically examined regulatory networks coupled with chromatin states and identified significant chromatin state-modified network motifs in four cell lines. Our results highlight the importance of chromatin states in information-processing of network motifs, which will increase our understanding of cell-specific functions.

## Material and Methods

### Data source

#### ChIP-seq

A total of 269 ChIP-seq data sets for 140 human transcription factors (TFs) over four cell lines, including embryonic stem cells (H1-hESC), lymphoblastoid (GM12878), myelogenous leukemia (K562) and liver carcinoma (HepG2), were used (Supplementary Table S4). Raw ChIP-Seq read data were obtained from the ENCODE Project Consortium (GSE32465 and GSE31477).

#### DNaseI hypersensitive sites

For each cell line, genome-wide DNase I hypersensitive sites (DHSs), which were identified using the HotSpot and peak-finding algorithms described in Sabo *et al*. (2004), were directly extracted from UCSC genome browser (DNaseI Hypersensitivity by Digital DNaseI from ENCODE/University of Washington (http://hgdownload.cse.ucsc.edu/goldenPath/hg19/encodeDCC/wgEncodeUwDnase/)^1^.

#### RNA-seq

We extracted RNA-seq data sets correspond to whole-cell long poly(A) + RNA for these four cell lines from ENCODE Cold Spring Harbor Laboratory (76-bp paired-end reads, GSE26284)^61^.

### Constructing transcriptional regulatory networks

For each ChIP-seq data set, raw reads were aligned to the human reference genome (hg19) using Bowtie (version 0.12.2), allowing up to two mismatches in the first 28-bp seed of the reads. Duplicate reads were removed and uniquely aligned reads were retained. Peaks were then called using MACS (version 1.4.2, *P*-value < 10-5). The 40-bp regions centered on the summit of peaks were used as TF binding sites (TFBS). To identify regulatory interactions, UCSC knownGene track (hg19) was downloaded from UCSC Genome Browser. Promoters were defined as a region of 1 kb around transcription start sites (TSS) with 0.5 kb upstream and 0.5 kb downstream.

Also, we obtained an average of 125,543 DHSs in the four cell lines. For each cell type, we filtered binding sites of each TF to those that followed into DHSs. A gene was considered as the target gene of a TF if at least one binding site of the TF located within the promoter region of the gene (Supplementary Table S5).

### Characterizing chromatin states at gene promoters

Genome-wide occupancy data for a set of multiple epigenetic marks were used to define 15 chromatin states based on recurrent combinations of marks across nine cell types using a multivariate Hidden Markov Model (HMM).We grouped these 15 states into four broad classes including strong activity, weak activity, poised state and repressed state.

Next, we characterized chromatin states at gene promoters through calculating fold enrichment between gene promoters and genomic regions with distinct chromatin states^1^ from the base level. Let *a*_*s*_ be the total number of bases in a given gene promoter with state *s*, *b* be the number of bases in the gene promoter, *c*_*s*_ be the total number of bases with state *s*, and *d* be the total number of nucleic acid base pairs (bp) marked by a specific chromatin state (such as ‘poised state’). The fold enrichment between the gene promoter and the state *s* was calculated by (*a*_*s*_/*b*)/(*c*_*s*_/*d*). By repeating the above procedure, we carried out fold enrichment calculation between the gene promoter and all of the four chromatin states, and chose the state with the maximum fold enrichment as the chromatin state of the promoter^3^.

### Identifying chromatin state-modified network motifs

For each transcriptional regulatory network, we assigned the chromatin states to the nodes (i.e., TFs and genes) for forming the regulatory network coupled with chromatin states. We then used FANMOD algorithm^62^ to identify chromatin state-modified three-node network motifs. The randomized networks that were used to calculate the significance of three-node subgraphs were generated to keep the same number of appearances of all two-node subgraphs as in the real network, which can avoid assigning a high significance to a network pattern only because it contains a highly significant subpattern. A total of 500 random networks were generated by iterative interaction swapping such that, apart from the same in- and out-degrees of each node are preserved, the ‘state-modified’ degree distribution of targets for each TF is separately preserved as the real network^63^,^64^,^65^. identified overrepresented motifs using a threshold of *P*-value < 0.05. For each type of state-modified motif, we also used a *P*-value which is the probability that a motif appears in 500 random networks an equal or greater number of times than in the real network, to evaluate the significance as previously proposed^63^,^64^,^65^. Subsequently, we identified significant state-modified network motifs according to the following criteria: (i) *P*-value < 0.05. (ii) The number of appearances in the real network (*N*real) larger than the average number of appearances in the random networks (*N*rand): *N*real– *N*rand > 0.05*N*rand^63^,^64^,^65^.

### Mutual exclusivity test

To assess the significance of mutually exclusive distribution of chromatin state composition coupled with network motifs, we used a column permutation method described by Bredel *et al*.^66^. The permutation-based approach calculates the probabilistic fit for mutual exclusivity of distribution of chromatin state compositions. A binary matrix *M* represents a distribution of chromatin state compositions (row, *i*) across different motif structures (column, *j*). The mutual exclusivity of distribution of appearance events within *M* was assessed based on a score *S*_*M*_:


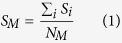


Where *N*_*M*_ represents the total number of chromatin state compositions which are significantly coupled with network motifs.


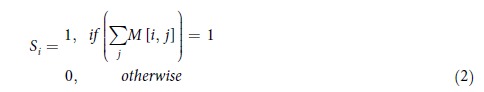


Where *S*_*i*_indicated whether the *i*-th chromatin state composition was coupled with only one motif structure. A set of 10,000 permutations were performed within the columns of the *M* to estimate the *P*-value. The *P*-value was determined as the fraction of permutations that lead to a greater or equal *S*_*M*_ score than that observed on real data.

### State transition test

To assess the significance of the observed state transitions in each position of FFL, we divided all FFL instances in the source cell line into different groups according to the alterations of FFL structures (including edge gain, loss or no change). For FFL instances in a given group, we calculated the number of FFL instances with a specific type of state transitions in a specific position of FFLs. We performed a permutation analysis to calculate the significance levels. In detail, we assembled artificial FFL instances using randomly selected TFs and targets from the regulatory network, keeping the same number of TFs and targets as observed in the real FFL instances. This process was then repeated 1,000 times to generate 1,000 artificial FFL instance sets in each comparison (H1 vs. GM12878 and GM12878 vs. K562). For each position, we computed the percentage of artificial FFL instance sets that showed higher frequency of state transition than the real FFL instance set as the *P*-value.

### Prioritization of candidate genes based on chromatin state and structure alterations of motifs

For a candidate gene, we extracted all chromatin state-modified FFL instances referring to this gene in cancer or normal cell line (i.e., K562 or GM12878). First, we quantified the chromatin state changes, for three components of each motif instance, between cancer and normal cell lines using a specific scoring matrix. A high score was given when chromatin state change seriously affected gene expression. Three criteria were used to produce the scoring matrix: I) ones with chromatin state changes from activation (e.g., strong and weak activity states) to repression (e.g., poised and repressed states) or vice versa were assigned the highest score of 10; II) ones with transition of the bivalent chromatin modification (that is, poised state) to the repressed state or vice versa were assigned a relatively low score of 5; III) ones with similar chromatin state transitions (e.g., strong and weak activity) were assigned the lowest score of 3. Next, a score of 5 was given if a motif showed structural changes (such as, gain or loss of edges) between cancer and normal cell lines. Third, the sum of scores (termed summary scores) from chromatin state and structural changes across all motif instances was used to assess the degree of changes, from the epigenetic and topological perspectives, between cancer and normal cell lines. The candidate genes with high summary scores indicate their potential effects on the development of cancer, relative to those with low scores. Finally, the summary scores were used to rank these candidate genes.

### Cell type-specific score

For a gene *i* in a specific cell line *j*, a cell type-specific score *S*_*ij*_ is calculated as its expression level divided by the total expression across all cell lines^67^:


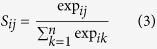


where *n* is the number of cell lines and *exp*_*ij*_ is the expression level of the *i*-th gene in *j*-th cell line. Genes with *S* > 0.85 were considered as cell type-specific genes^68^.

### Functional enrichment analysis

Gene Ontology (GO) analysis of genes was performed using GOstats package from Bioconductor. Significantly enriched GO terms were identified by hypergeometric test:


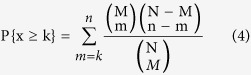


where *N* represents the size of the background, *n* represents the size of a GO term, *M* represents the number of the genes of interest, *k* represents the number of genes annotated with the GO term. For different types of chromatin state-modified FFLs, we identified all instances in the regulatory networks, which should satisfy: 1) the FFL structure; 2) the specific chromatin state composition. Thus we used all genes in the regulatory networks as background to perform functional enrichment analysis. Only GO terms at levels below 4 were used. A FDR-corrected *P* of 0.05 was used as the threshold for significantly enriched GO categories.

## Additional Information

**How to cite this article**: Zhao, H. *et al*. Chromatin states modify network motifs contributing to cell-specific functions. *Sci. Rep*. **5**, 11938; doi: 10.1038/srep11938 (2015).

## Supplementary Material

Supplementary Information

Supplementary Table S5
