# Supplementary material for: Chromatin states modify network motifs contributing to cell-specific functions
Source: Sci Rep. 2015 Jul 14;5:11938. doi: 10.1038/srep11938 (PMC4500950; doi:10.1038/srep11938)
Supplement: Supplementary Information [file srep11938-s1.pdf]

Chromatin states modify network motifs contributing to cell-specific functions

Hongying Zhao[1], Tingting Liu[1], Ling Liu[1], Guanxiong Zhang[1], Lin Pang[1], Fulong Yu[1], Huihui Fan[1], Yanyan Ping[1], Li Wang[1], Chaohan Xu[1], Yun Xiao[1,2,*], Xia Li[1,*]

[*]To whom correspondence should be addressed.

[1]College of Bioinformatics Science and Technology, Harbin Medical University, Harbin 150081, China.

[2]Key Laboratory of Cardiovascular Medicine Research, Harbin Medical University, Ministry of Education.

[*]**Correspondening Authors:**

Dr. Xia Li, College of Bioinformatics Science and Technology, Harbin Medical University, No.194, Xue-Fu road, Nangang region, Harbin 150081, China (Email: lixia@hrbmu.edu.cn). Phone: 86-451-86615922; Fax: 86-451-86615922.

Dr. Yun Xiao, College of Bioinformatics Science and Technology, Harbin Medical University, No.194, Xue-Fu road, Nangang region, Harbin 150081, China (Email: xiaoyun@ems.hrbmu.edu.cn).

Supplementary methods

The workflow (Supplementary Fig. S8) for constructing chromatin state-modified regulatory networks by integrating distal binding sites of TFs and chromatin states from the HMM approach. The detailed description of these steps is given below:

Step 1: Defining TFBS, enhancer, promoter and distal regions

(a) TF binding sites (TFBS) were detected by ChIP-seq in four cell lines (including H1, GM12878, K562, HepG2). Those TFBSs which followed into DNase I hypersensitive sites (DHS) were retained for the following analysis.

(b) Promoters were defined as a region of 1 kb around transcription start sites (TSS) with 0.5 kb upstream and 0.5 kb downstream. The genome regions from -100kb to -10 kb and from +10kb to +100kb regions of TSS were considered as the distal region of a gene[1-3].

(c) ChIP-seq data sets of histone marks H3K4me1 and H3K4me3 from H1 cell line were obtained and peaks were called using MACS (version 1.4.2, P-value< 10-5). Then, we identified the enhancers based on the chromatin signature pattern: the presence of H3K4me1 but absence of H3K4me3[4,5].

Step 2: Identifying target genes of an enhancer

The target genes of enhancers were identified as follow: (a) we calculated the intensities of pol2 and H3K27ac at promoters and the intensities of H3K4me1 and H3K27ac at enhancers in four cell lines. (b) For a promoter with at least one enhancer located in its distal region, we calculated the Spearman correlation coefficient (SCC) between the promoter and its distal enhancers (SCC_1: pol2 at promoter vs H3K4me1 at enhancer, SCC_2: H3K27ac at promoter vs H3K27ac at enhancer), respectively. (c) the promoter-enhancer interaction was identified if both SCC_1 and SCC_2 were above 0.23[6].

Step 3: Identifying the TF-gene interactions mediated by distal TF binding: if a distal TF binding site located in an enhancer region, targets of the enhancer were regarded as the targets of the TF.

Step 4: Identifying promoter-mediated regulatory interactions: if at least one

binding site of the TF located within the promoter region of a gene, the gene was considered as a target gene of the TF.

Step 5: constructing regulatory network by integrating promoter- and enhancer-mediated regulatory interactions.

Step 6: Identifying chromatin states of genes based on the HMM approach: using a four-state hidden Markov model (HMM) approach, a seven-dimensional histone modification profile corresponding to the RPKM values of epigenetic marks over the promoter (for H3K4me1, H3K4me2, H3K4me3, H3K9me3, H3K27ac and H3K27me3) and coding region (for H3K36me3) was used to characterize chromatin states of each gene in H1 cell line[7].

Step 7: Construct chromatin state-modified regulatory network by integrating the regulatory network and chromatin states of genes.

Supplementary Figures

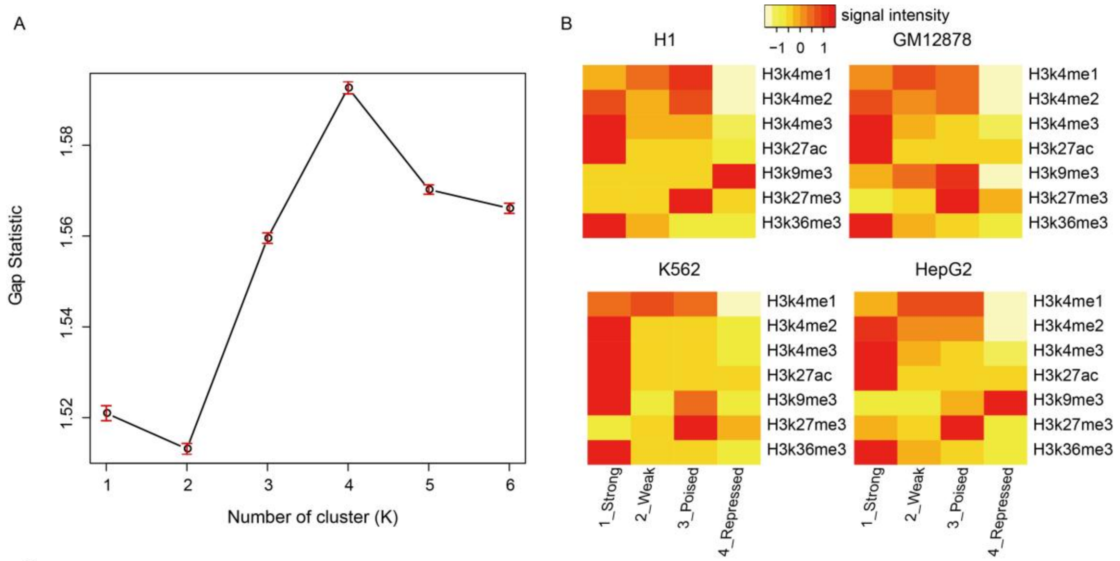

C

H1	H3k4me1	H3k4me2	H3k4me3	H3k27ac	H3k9me3	H3k27me3	H3k36me3
2_Weak_Active.VS.1_Strong_Active	1.61E-112	0.00E+00	0.00E+00	0.00E+00	0.036994	9.80E-106	0.00E+00
2_Weak_Active.VS.3_Poised_State	2.41E-46	1.59E-269	1.82E-19	2.07E-245	0.093358	0.00E+00	5.92E-58
GM12878	H3k4me1	H3k4me2	H3k4me3	H3k27ac	H3k9me3	H3k27me3	H3k36me3
2_Weak_Active.VS.1_Strong_Active	4.26E-72	9.33E-71	0.00E+00	0.00E+00	6.13E-05	5.26E-208	0.00E+00
2_Weak_Active.VS.3_Poised_State	2.09E-15	3.58E-02	1.96E-21	6.59E-143	1.20E-04	0.00E+00	8.20E-38
K562	H3k4me1	H3k4me2	H3k4me3	H3k27ac	H3k9me3	H3k27me3	H3k36me3
2_Weak_Active.VS.1_Strong_Active	0.02305709	0	0	0.00E+00	3.56E-43	3.47E-39	0
2_Weak_Active.VS.3_Poised_State	0.00012966	0.9180899	0.3651228	3.48E-28	1.44E-04	2.60E-129	0.005467
HepG2	H3k4me1	H3k4me2	H3k4me3	H3k27ac	H3k9me3	H3k27me3	H3k36me3
2_Weak_Active.VS.1_Strong_Active	1.83E-93	0	0.00E+00	0.00E+00	0.411887	3.37E-131	0.00E+00
2_Weak_Active.VS.3_Poised_State	3.63E-03	0.0002581	7.91E-16	4.56E-47	0.017041	1.32E-256	3.13E-20

Supplementary Figure S1. Assessment of the optimal number of chromatin state categories. (A) Gap statistic showing that K=4 is the optimal number. The signal intensities of diverse epigenetic marks (B) and the statistical significance from Wilcoxon rank-sum test (C) of different epigenetic marks between different chromatin state categories were shown in four cell lines.

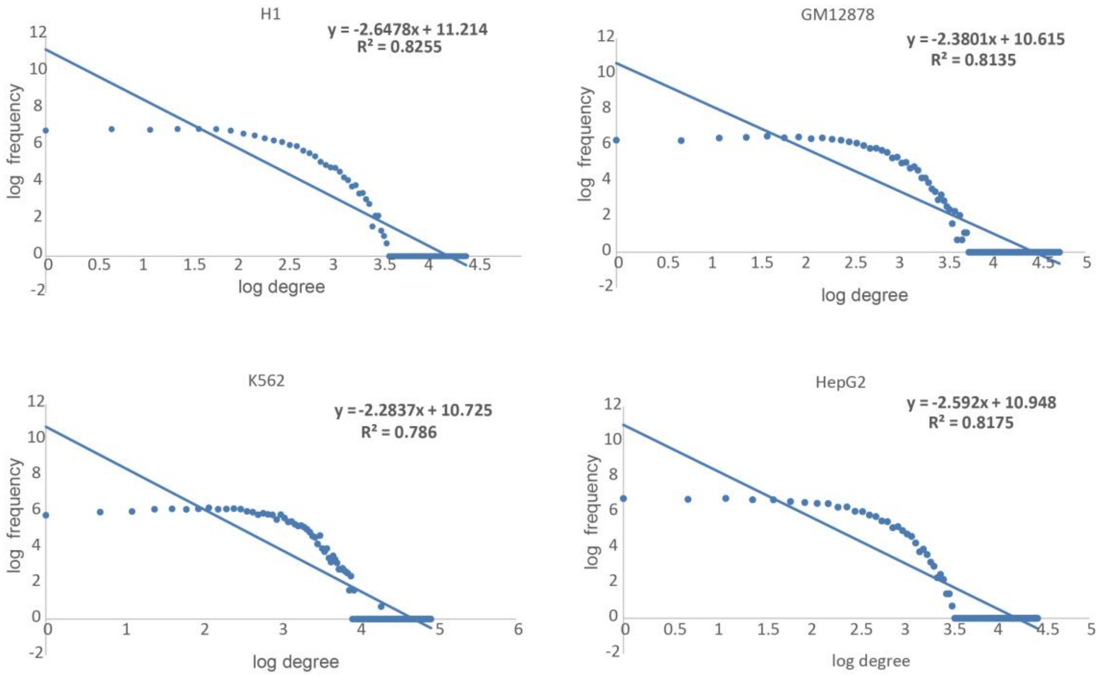

Supplementary Figure S2. The degree distribution of the transcriptional regulatory networks in four cell lines. Horizontal axis represents log10 transformed degree, and vertical axis represents log10 transformed frequency. The degree distribution for each cell line follows a power law (R^2=0.83, 0.81, 0.79 and 0.82).

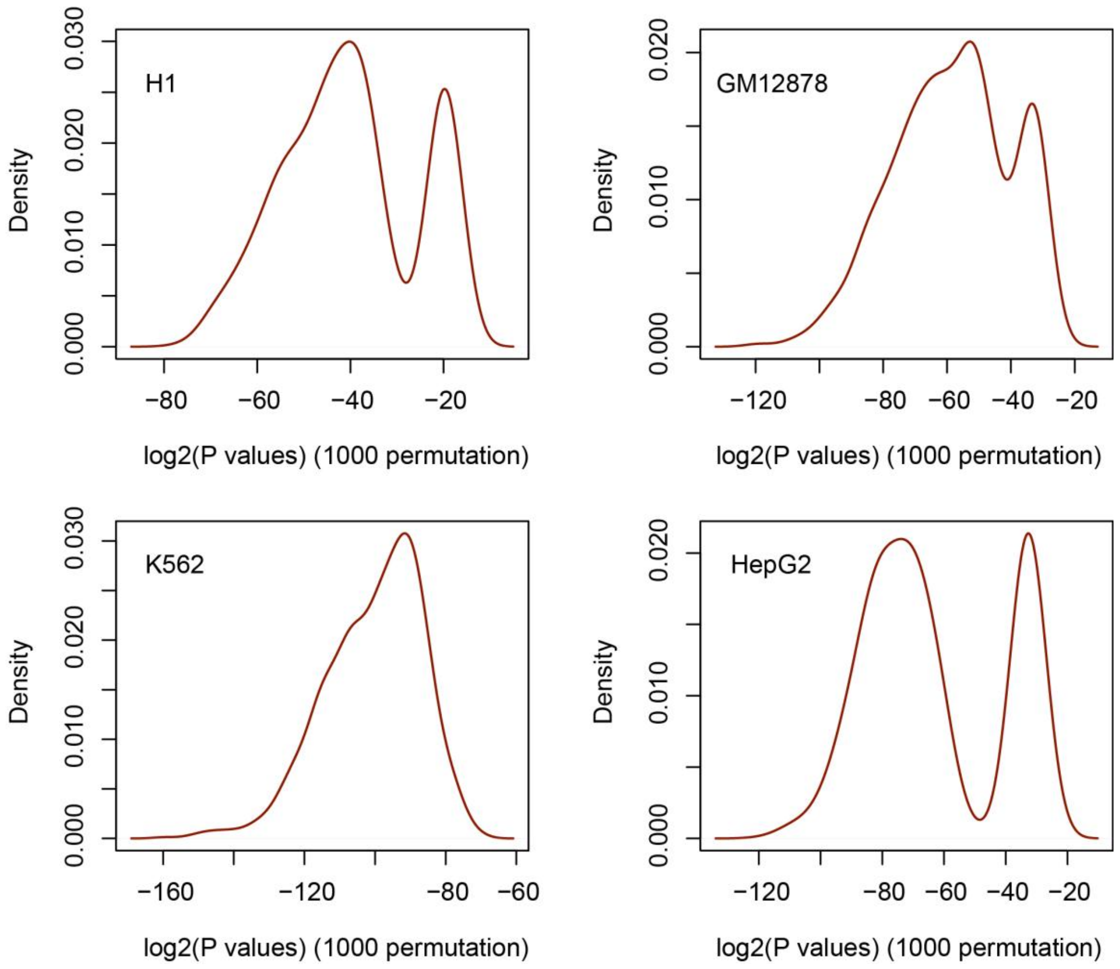

Supplementary Figure S3. Significant difference in expression level between genes with ‘weak activity’ state and the mixture of other groups. Wilcoxon rank-sum test was used to compare expression levels of group 2 (weak activity state) with the same size of an artificial gene set randomly selected from a mixture of group 1 (strong activity state) and group 3 (poised state). This process was repeated 1,000 times, resulting in a distribution of *p* values for each cell line.

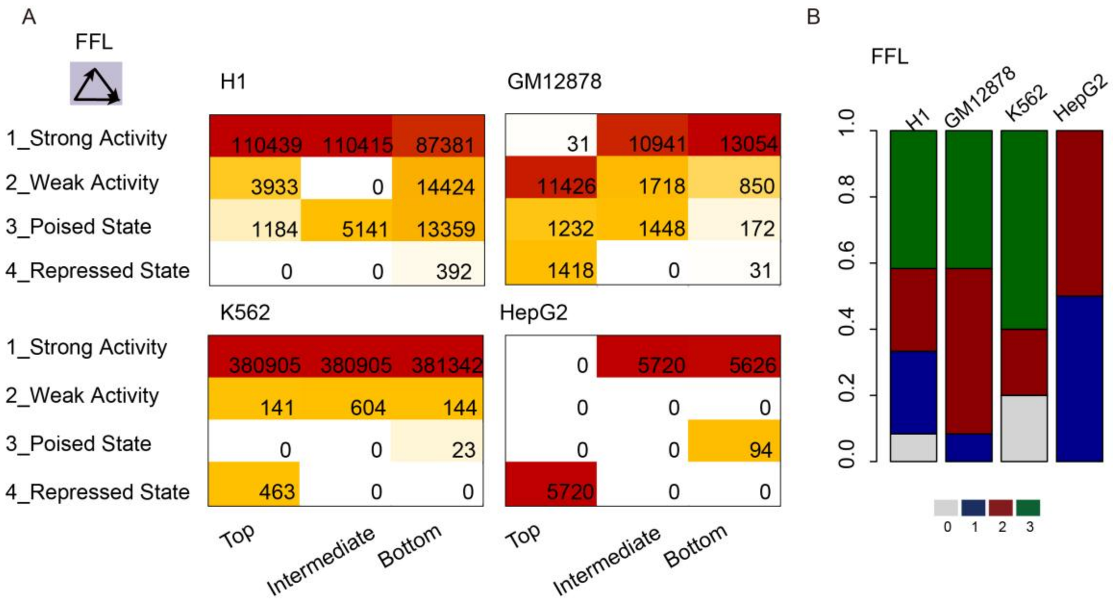

Supplementary Figure S4. The distribution of chromatin state compositions coupling with network motifs in four cell lines. (A)The distribution of the numbers of chromatin state compositions associated with a particular motif in four cell lines in the order of top, intermediate and bottom positions. The values indicate the frequency of motif instances. And rows indicate four chromatin states used in this study. (B) The frequency of changes in significantly enriched chromatin state compositions associated with FFL in four cell lines when comparing with the major fully active state. Changes of chromatin states in zero (gray), one (blue), two (red) and three (green) positions were showed.

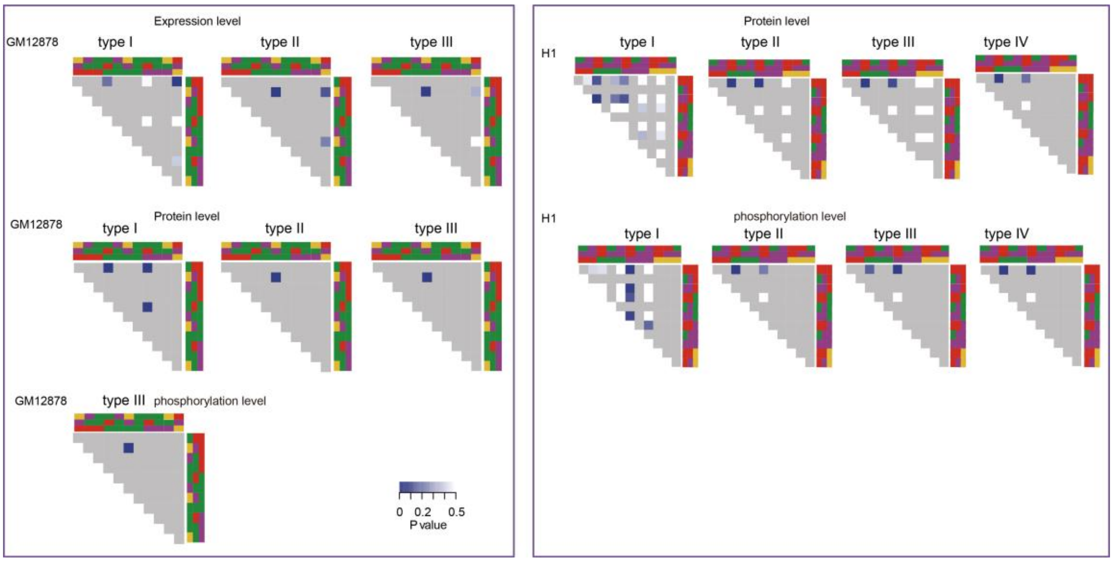

Supplementary Figure S5. The difference of target genes in expressions, protein and phosphorylation levels within each type of comparable FFLs in four cell lines. For each cell line, the comparisons in three levels of the target genes across different chromatin state compositions in the same type of FFLs were shown, and no comparable FFLs were found in K562 and HepG2. Color intensities (blue) correspond to *P*-values of Wilcoxon rank-sum test results. Gray entries represent data unavailable. Colored rectangles indicated the chromatin states of genes at top, intermediate and bottom position in FFLs.

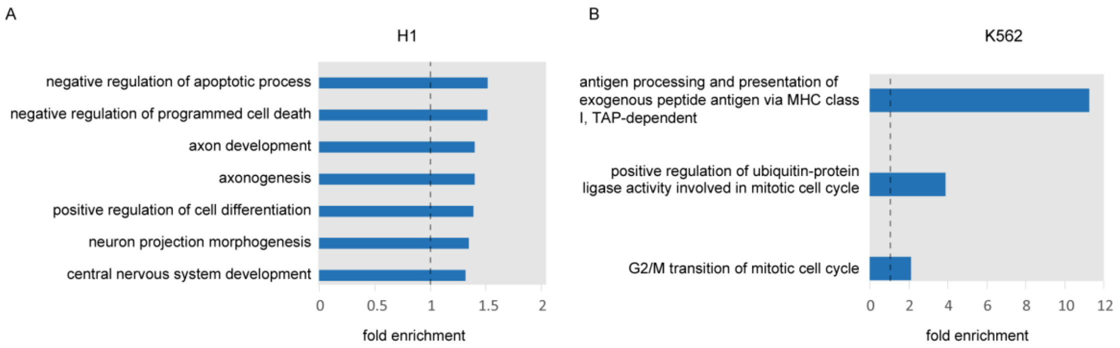

Supplementary Figure S6. Functional enrichment analysis using specific background gene sets. (A) The significant biological processes enriched by the FFL target poised genes using all poised genes of H1 network as background. (B) The significant biological processes enriched by 'strong activity' genes targeted by the 'all-strong-activity' FFL using all 'strong activity' genes of K562 network as

background.

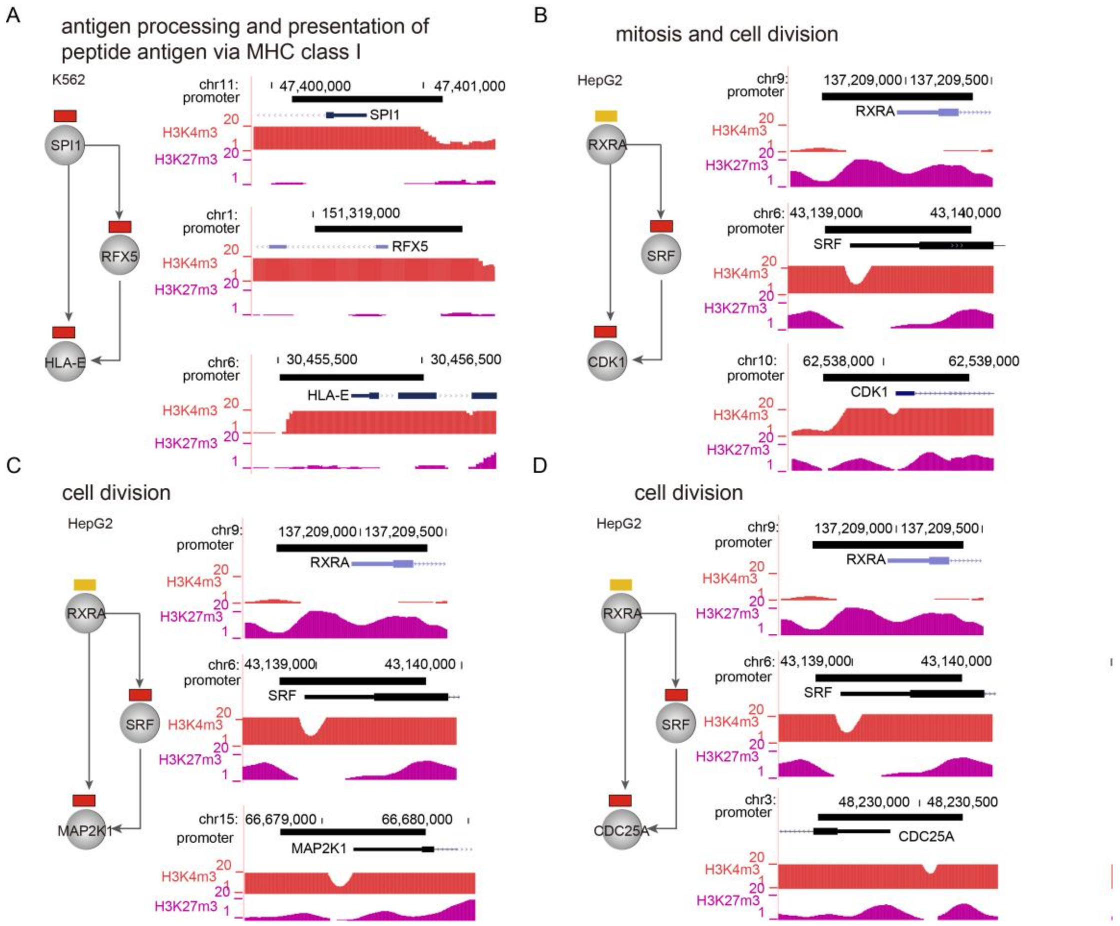

Supplementary Figure S7. Examples of chromatin-state modified FFLs enriched in different biological processes and their associated H3K4me3 (red) and H3K27me3 (purple) distributions in K562 and HepG2.

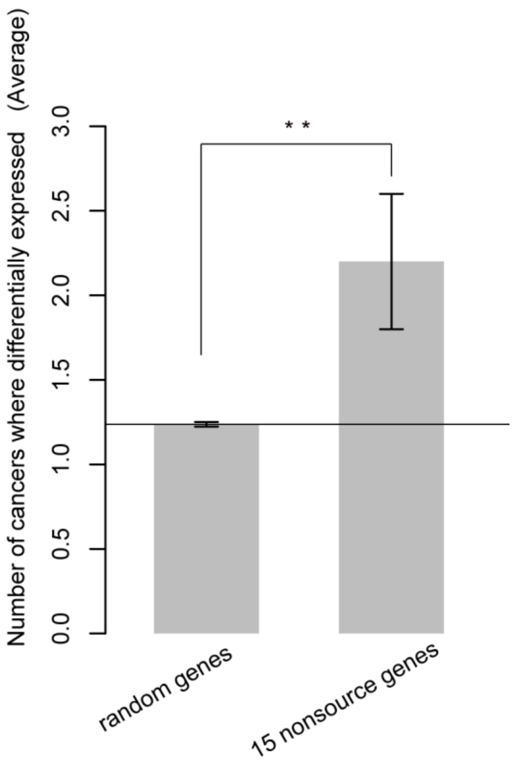

Supplementary Figure S8. Dysregulation of top-ranked ‘non-source-node’ genes in diverse cancer types. The average number of 12 TCGA cancer types in which top-ranked ‘non-source-node’ genes differentially expressed. ** represents P-value<0.001.

A

H1	Active	Weak	Poised	Repressed
H3k4me1	1.7722	2.2122	2.9011	0.63739
H3k4me2	15.98	1.6434	15.1	0.48892
H3k4me3	25.097	1.1151	10.679	0.599
H3k27ac	6.5033	1.3288	1.1547	0.70801
H3k9me3	0.40871	0.54831	0.45194	0.97005
H3k27me3	0.80461	1.0727	10.393	0.74627
H3k36me3	2.5324	1.4204	0.87821	0.63855

GM12878	Active	Weak	Poised	Repressed
H3k4me1	0.66939	1.7778	0.98816	0.21839
H3k4me2	6.1494	7.584	3.7969	0.26426
H3k4me3	4.3026	2.9084	1.0126	0.1689
H3k27ac	10.727	4.7628	0.39686	0.1283
H3k9me3	0.37046	0.5375	0.57629	0.3097
H3k27me3	0.52784	1.1778	2.8109	1.3598
H3k36me3	1.6919	1.1041	0.31117	0.29684

K562	Active	Weak	Poised	Repressed
H3k4me1	0.98457	1.7574	1.5821	0.31978
H3k4me2	11.103	3.6658	19.404	0.38029
H3k4me3	8.092	2.3518	14.277	0.29336
H3k27ac	7.6421	1.2592	16.587	0.19165
H3k9me3	0.28221	0.39275	0.55447	0.29873
H3k27me3	0.22742	0.46734	0.42704	0.53308
H3k36me3	1.2096	0.80701	1.9411	0.22654

HepG2	Active	Weak	Poised	Repressed
H3k4me1	1.3339	3.9874	2.7342	0.46835
H3k4me2	15.752	25.004	7.7036	0.53902
H3k4me3	39.956	50.58	9.0923	0.52198
H3k27ac	20.319	38.153	1.1753	0.18265
H3k9me3	0.20711	0.4453	0.26495	0.46883
H3k27me3	1.7033	2.8608	1.8888	1.2665
H3k36me3	3.5831	5.1492	1.4994	0.32419

B

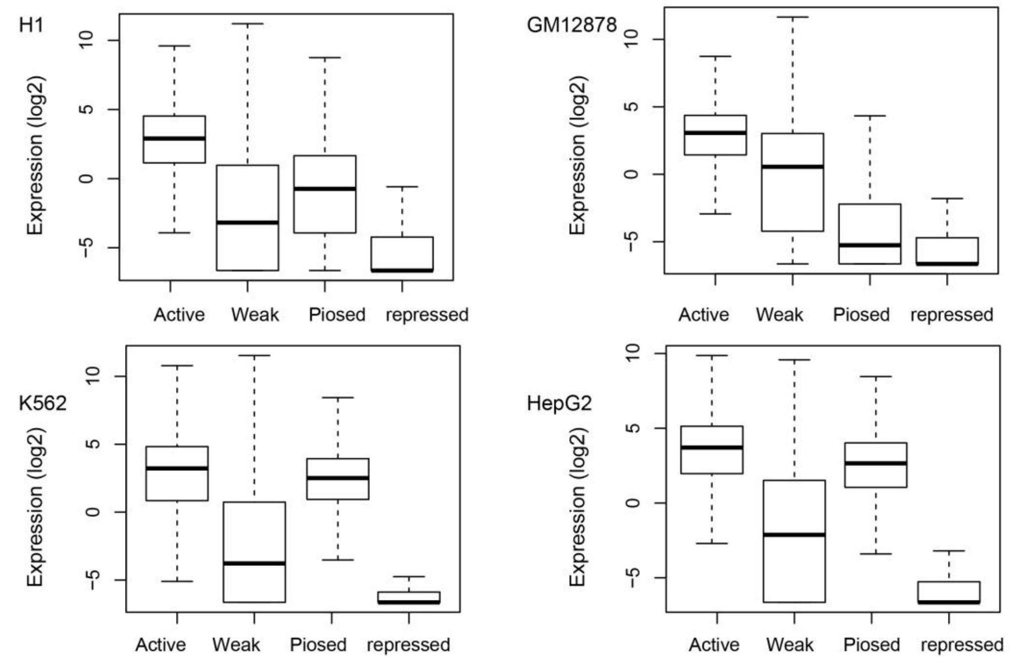

Supplementary Figure S9. The average histone modification levels (A) and gene expression levels (B) within each state category from the HMM approach. Red (or green) colors represent high (or low) histone modification levels.

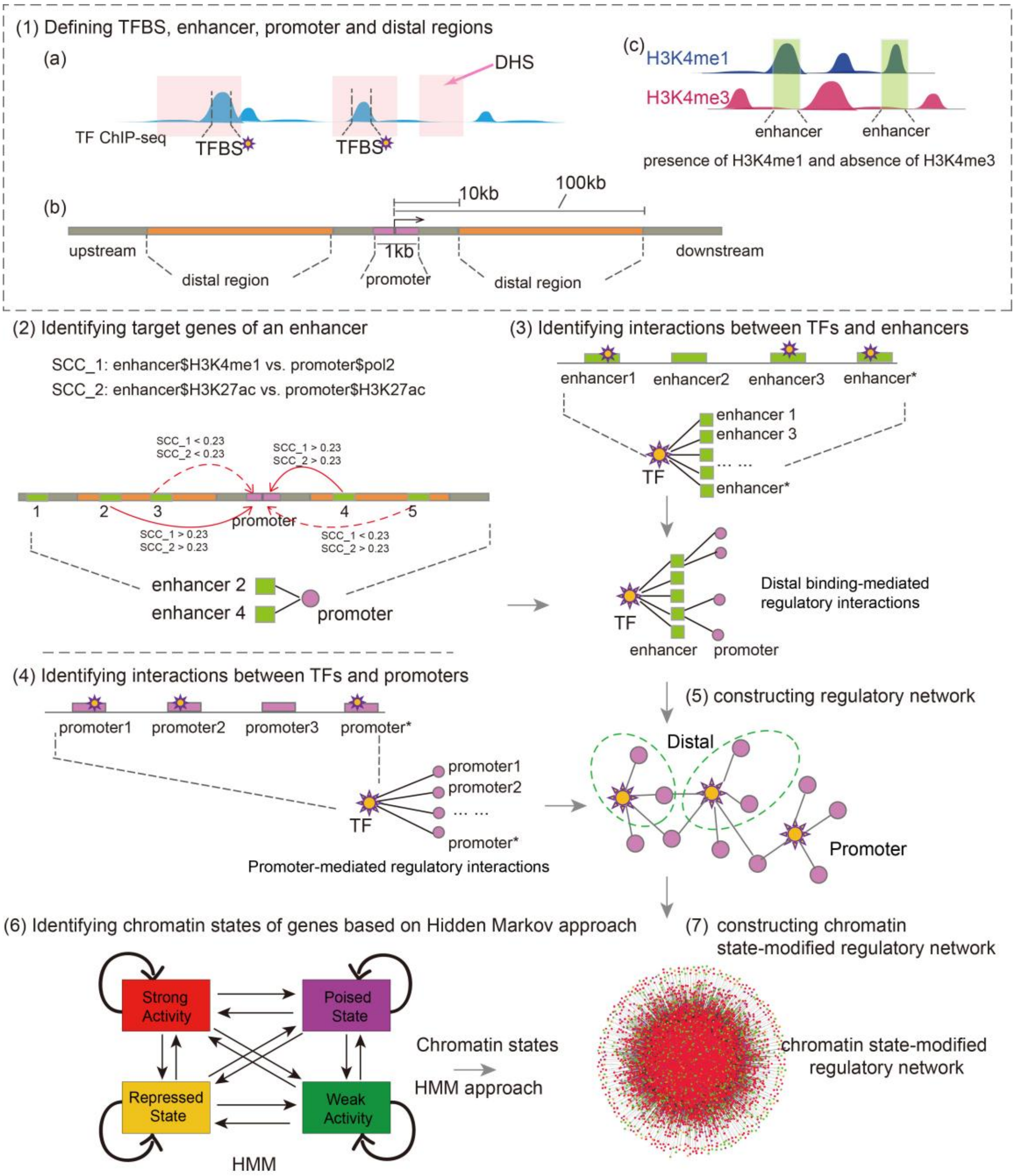

Supplementary Figure S10. Workflow for constructing chromatin state-modified transcription regulatory networks by combining proximal and distal regulations and the chromatin states obtained from a four-state HMM approach.

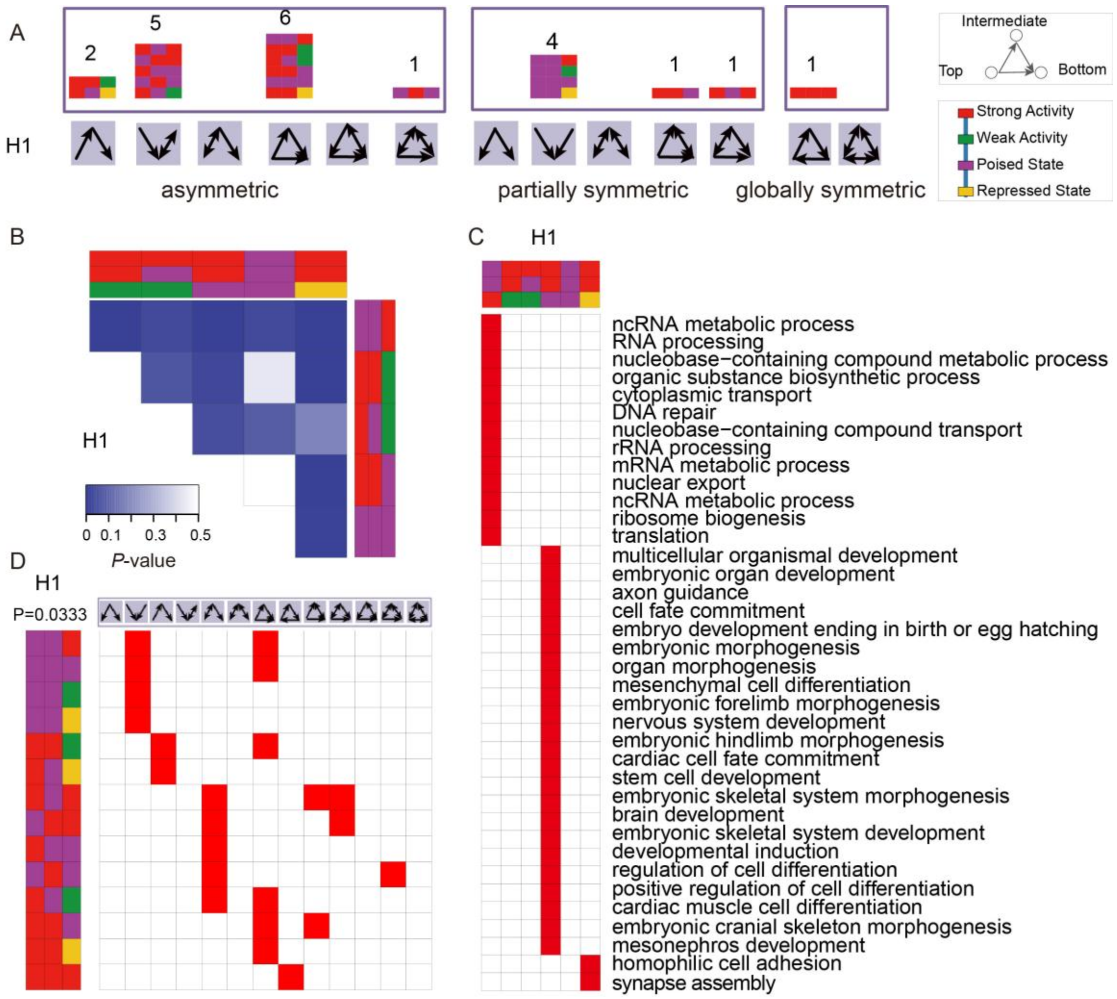

Supplementary Figure S11. Results of the approach with considering the distal regulatory regions of a gene. (A) Over-represented chromatin state compositions associated with network motifs in H1. Asymmetric, partially and globally symmetric motifs are shown from left to right. For the symmetric motifs, only one of possible combinations of chromatin states was displayed. Chromatin states of gene promoter in each type of motif are shown in the order of top, intermediate and bottom positions. Values are presented as the number of chromatin state compositions. (B) Expression differences between targets of different chromatin state-modified FFLs. Colored rectangles indicated the chromatin states of genes at top, intermediate and bottom position in FFLs. (C) The significant biological processes enriched by target genes of each chromatin state-modified FFL. (D) The distribution of chromatin state compositions across thirteen types of FFLs.

cellLines	TFs	edges	degree	diameter	betweenness
H1	48	103451	16.5	6	55.9
GM12878	79	111407	20.9	6	79.1
K562	88	151589	28.4	6	95.6
HepG2	53	94509	16.9	8	54.2

Supplementary Table S1. The topological properties of chromatin state-modified networks in four cell lines.

Activators (29)	Repressors (28)	Dual regulators (46)	Unkown (37)
ATF2 NRF1 TAF1 TBP	BCLAF1 SIN3A EBF1	KAT2A STAT3 YY1 ATF3 ESRRA	CHD1 WRNIP1 ZZZ3 GABPB2
BCL3 FOXM1 NFATC1	MXI1 NFIC ZEB1 BATF	GABPA RUNX3 BRCA1 CHD2	MAFK NFYB PBX3 RAD21
CEBPB CREB1 EGR1	BCL11A BHLHE40 E2F4	CTCF CUX1 ELF1 IRF4 JUND	SMC3 ZNF384 GTF2F1
ELK1 EP300 ETS1 FOS	IKZF1 RCOR1 REST RXRA	MAX MEF2A MTA3 MYC	TEAD4 ZNF274 MAFK
IRF3 MAZ MEF2C	SUPT20H TCF12 TCF3	NR2C2 PAX5 PML POU2F2	ARID3A BDP1 BRF1 CBX5
NFE2 NFYA SPI1	BACH1 CTBP2 E2F6	RFX5 SIX5 SP1 SREBF1 SRF	CCNT2 GTF2B GTF3C2
SREBF2 ZNF143	FOSL1 HDAC2 SUZ12	STAT5A USF1 USF2 ZBTB33	HCFC1 HMGN3 MAFF NELFE
GABPB1 JUN TAF7	CTCFL HEY1 SIRT6	YY1 NANOG POU5F1 SP2 SP4	POLR3A THAP1 SETDB
CEBPD SMARCA4	TRIM28 FOXA2	ATF1 GATA1 GATA2 NR2F2	TBL1XR1 UBTF XRCC4
SMARCB1 FOXA1		ZBTB7A TAL1 CEBPZ FOSL2	ZC3H11A ZFP36L2 ZNF263
		HNF4A TCF7L2	HNF4G MBD4 MYBL2

Supplementary Table S2. The list of activators, repressors, dual regulators and indeterminate TFs involved in the current study.

Supplementary Table S3. Ranked gene list by integrating alterations of chromatin states and motif structures. (A) Gene list including 67 ‘source-node’ genes (the TFs with ChIP-seq data available in the corresponding cell type) and 15 ‘non-source-node’ genes. (B) All ranked gene list.

	GM12878	H1	K562	HepG2
TF ChIP-seq	ATF2,ATF3,BATF,BCL11A,BCL3,BCLAF1,BHLHE40,BRCA1,CEBPB,CHD1,CHD2,CREB1,CTCF,CUX1,E2F4,EBF1,EGR1,ELF1,ELK1,EP300,ESRRA,ETS1,FOS,FOXM1,GABPA,IKZF1,IRF3,IRF4,JUND,KAT2A,MAFK,MAX,MAZ,MEF2A,MEF2C,MTA3,MXI1,MYC,NFATC1,NFE2,NFIC,NFYA,NFYB,NR2C2,NRF1,PAX5,PBX3,PML,POU2F2,RAD21,RCOR1,REST,RFX5,RUNX3,RXRA,SIN3A,SIX5,SMC3,SP1,SPI1,SREBF1,SREBF2,SRF,STAT1,STAT3,STAT5A,SUPT20H,TAF1,TBL1XR1,TBP,TCF12,TCF3,USF1,USF2,WRNIP1,YY1,ZBTB33,ZEB1,ZNF143,ZNF274,ZNF384,ZZZ3	ATF2,ATF3,BACH1,BCL11A,BRCA1,CEBPB,CHD1,CHD2,CREB1,CTBP2,CTCF,E2F6,EGR1,EP300,FOSL1,GABPA,GTF2F1,HDAC2,JUN,JUND,AFK,MAX,MXI1,MYC,NANOG,NRF1,POU5F1,RAD21,REST,RFX5,RXRA,SIN3A,SIX5,SP1,SP2,SP4,SRF,SUZ12,TAF1,TAF7,TBP,TCF12,TEAD4,USF1,USF2,YY1,ZNF143,ZNF274	ARID3A,ATF1,ATF3,BACH1,BCL3,BCLAF1,BDP1,BHLHE40,BRF1,CBX5,CCNT2,CEBPB,CEBPD,CHD2,CREB1,CTCF,CTCFL,CUX1,E2F4,E2F6,EGR1,ELF1,ELK1,EP300,ETS1,FOS,FOSL1,GABPA,GATA1,GATA2,GTF2B,GTF2F1,GTF3C2,HCFC1,HDAC2,HEY1,HMGN3,JUND,JUN,MAFF,MAFK,MAX,MAZ,MEF2A,MXI1,MYC,NELFE,NFE2,NFYA,NFYB,NR2C2,NR2F2,NRF1,PML,RAD21,RCOR1,REST,RFX5,SETDB1,SIRT6,SIX5,SMARCA4,SMARCB1,SMC3,SP1,SP2,SPI1,SRF,STAT5A,TAF1,TAF7,TAL1,TBL1XR1,TBP,TEAD4,THAP1,TRIM28,UBTF,USF1,USF2,XRCC4,YY1,ZBTB33,ZBTB7A,ZC3H11A,ZFP36L2,ZNF143,ZNF263,ZNF274,ZNF384	ARID3A,ATF3,BHLHE40,BRCA1,CEBPB,CEBPD,CEBPZ,CHD2,CREB1,CTCF,ELF1,EP300,FOSL2,FOXA1,FOXA2,GABPA,HDAC2,HEY1,HNF4A,HNF4G,IRF3,JUND,JUN,MAFF,MAFK,MAX,MAZ,MBD4,MYBL2,NFIC,NR2C2,NR2F2,NRF1,RAD21,RCOR1,REST,RFX5,RXRA,SMC3,SP1,SP2,SRF,TAF1,TBP,TCF12,TCF7L2,TEAD4,USF1,USF2,YY1,ZBTB33,ZBTB7A,ZEB1,ZNF274
	79TFs	48TFs	88TFs	54TFs
DNase-seq	ENCODE/University of Washington<br>http://hgdownload.cse.ucsc.edu/goldenPath/hg19/encodeDCC/wgEncodeUwDnase			

Supplementary Table S4. Cell lines, transcription factors and DNase-seq involved in the current study.

Cell type	Filtering method			ChIP-seq TF-gene	Overlap -w TRANSFAC	Precision $^{0}/_{00}$
H1	-w/o DNase			254552	472	1.854238
	w/ DNase	Hotspots	Inside	244516	450	1.840370
			Overlap	247428	456	1.842960
		Narrow peak	Inside	103576	250	**2.413687**
			Overlap	154843	328	2.118275
GM12878	-w/o DNase			261986	521	1.988656
	w/ DNase	Hotspots	Inside	252954	488	1.929205
			Overlap	254486	493	1.937238
		Narrow peak	Inside	112120	255	**2.274349**
			Overlap	165481	363	2.193605
K562	-w/o DNase			393678	448	1.137986
	w/ DNase	Hotspots	Inside	23505	31	1.318868
			Overlap	28160	36	1.278409
		Narrow peak	Inside	154352	211	**1.367005**
			Overlap	229027	288	1.257494
HepG2	-w/o DNase			229848	428	1.862100
	w/ DNase	Hotspots	Inside	223331	399	1.786586
			Overlap	224685	401	1784721
		Narrow peak	Inside	94595	211	**2.230562**
			Overlap	143921	292	2.028891
Mean	-w/o DNase			285016.0	467.25	1.710745
	w/ DNase	Hotspots	Inside	186076.5	342.00	1.718757
			Overlap	188689.8	346.50	1.710832
		Narrow peak	Inside	116160.8	231.75	**2.071401**
			Overlap	173318.0	317.75	1.899566

Supplementary Table S5. Evaluation of TF-gene interactions using ChIP-seq and DNase-seq. TF-gene interactions were filtered out using different criterions, including TF binding site locating within promoter without using DNase I hypersensitive sites (DNase peaks; -w/o); TF binding site locating within promoter inside or overlapping with Hotspots or DNase narrow peaks.

References

1. Adachi, K. et al. Context-dependent wiring of Sox2 regulatory networks for self-renewal of embryonic and trophoblast stem cells. *Mol Cell* **52**, 380-92 (2013).
2. Kanhere, A. et al. T-bet and GATA3 orchestrate Th1 and Th2 differentiation through lineage-specific targeting of distal regulatory elements. *Nat Commun* **3**, 1268 (2012).
3. Levine, M. & Tjian, R. Transcription regulation and animal diversity. *Nature* **424**, 147-51 (2003).
4. Heintzman, N.D. et al. Distinct and predictive chromatin signatures of transcriptional promoters and enhancers in the human genome. *Nat Genet* **39**, 311-8 (2007).
5. Heintzman, N.D. et al. Histone modifications at human enhancers reflect global cell-type-specific gene expression. *Nature* **459**, 108-12 (2009).
6. Shen, Y. et al. A map of the cis-regulatory sequences in the mouse genome. *Nature* **488**, 116-20 (2012).
7. Larson, J.L. & Yuan, G.C. Epigenetic domains found in mouse embryonic stem cells via a hidden Markov model. *BMC Bioinformatics* **11**, 557 (2010).
